# Projected Methane Emissions From a Boreal Thermokarst Bog Are Sensitive to Carbon Substrate Availability, Distribution, and Transport Pathway Dominance

**DOI:** 10.1111/gcb.70880

**Published:** 2026-04-28

**Authors:** Benjamin C. Maglio, Ruth Rutter, Valeria Briones, Andrew Mullen, Chu‐Chun Chang, Joshua M. Rady, Tobey Carman, Colin Edgar, Eugénie S. Euskirchen, Jennifer D. Watts, Susan M. Natali, Elchin E. Jafarov, Brendan M. Rogers, Hélène Genet

**Affiliations:** ^1^ Institute of Arctic Biology University of Alaska Fairbanks Fairbanks USA; ^2^ Woodwell Climate Research Center Falmouth USA

**Keywords:** biogeochemical cycles, biological feedback, climate change, ecosystem modeling, greenhouse gases, permafrost

## Abstract

Methane is a potent greenhouse gas, which traps significantly more heat than carbon dioxide over short timescales, but remains underrepresented in models used for projecting future climate scenarios, particularly in permafrost landscapes. Understanding methane dynamics in northern wetlands is critical for improving projections of high‐latitude carbon feedbacks to the global climate system. To address this, we integrated ecological processes driving methane production, oxidation, and transport pathways into the DVM‐DOS‐TEM terrestrial biosphere model. Using long‐term site observations from a thermokarst bog in boreal Alaska, we calibrated and benchmarked the model against observations of methane and carbon dioxide fluxes, carbon and nitrogen stocks, and soil temperature and moisture. Parameter sensitivity analyses identified opposing correlations for methanogenesis and heterotrophic respiration, revealing a dependence on carbon availability. We also found sensitivity to the distribution of carbon relative to the water table position. Investigation of the dominance of different methane transport pathways demonstrated the need for observations of methane flux partitioning, and the utility in comparing simulated to observed seasonality. The cumulative methane efflux projected to the year 2100 had a range of 211–3470 g C m^−2^ between low and high warming scenarios. Methane emissions dominated by diffusion showed the greatest variability across projections. Increased soil temperature and carbon availability from permafrost thaw resulted in increasing methane emissions between 2030 and 2060. But methane emission was then limited by a deepening of the water table. Nevertheless, we estimated a positive radiative forcing (i.e., warming) from these mid‐century methane emissions that persisted until 2100. An ebullition‐dominant parameterization led to lower variability but a net negative radiative forcing (i.e., cooling) on average. Our study highlights the importance of representing methane emission pathways and the uncertainty associated with partitioning them on predicting the carbon budget and radiative forcing of wetland ecosystems in high latitudes.

## Graphical abstract

Projected radiative forcing from a thermokarst bog in Boreal Alaska depends on methane emission transport pathway dominance, with aerenchyma‐ and diffusion‐dominant causing net warming and ebullition‐dominant causing net cooling. Predicted methane shows a transient response as air temperature increases in future projections causing soil drying and lowering of the water table as permafrost thaws.

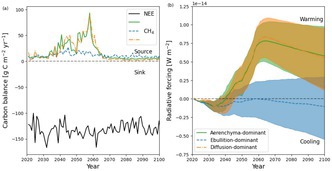

## 1 Introduction

The Arctic‐Boreal domain occupies ~22% of the terrestrial land surface (Chapin III et al. 2000) and is experiencing rates of warming up to four times greater than the rest of the planet (Rantanen et al. 2022). The northern permafrost region, located predominantly within these biomes, is estimated to hold more than twice the current atmospheric carbon in perennially frozen ground, or permafrost (Tarnocai et al. 2009; Hugelius et al. 2014; Schuur et al. 2022). Carbon release from permafrost thaw as greenhouse gases is likely to be one of the most significant positive feedbacks to the climate, that is, the permafrost carbon‐climate feedback (PCCF; common abbreviations are listed in the Supporting Information; Schaefer et al. 2014; Schuur et al. 2022). The magnitude and composition of carbon release in the form of carbon dioxide (CO_2_) or methane (CH_4_) is determined by local permafrost conditions and hydrology (Schuur et al. 2015; Olefeldt et al. 2013) and can impact the net radiative forcing resulting from permafrost thaw. Though current Arctic‐Boreal carbon emissions are dominated by CO_2_ (Miner et al. 2022), potential climate‐driven wetland expansion in the boreal region may increase CH_4_ production (methanogenesis) by the end of the century (Zhang et al. 2017).

CH_4_ is the second most abundant greenhouse gas in the atmosphere with a global warming potential 80–85 times higher than CO_2_ over 20 years (Wang et al. 2024). Though the majority (~60%) of global CH_4_ emissions have been attributed to anthropogenic sources, wetlands, which are the largest natural source (Ueyama et al. 2023), have been estimated to contribute around 25% of global emissions (Saunois et al. 2020) and account for the majority of recent increases in atmospheric CH_4_ (Forbrich et al. 2024). Methanogenesis is driven by soil temperature, organic matter availability, and redox conditions, which are modulated by vegetation composition, permafrost conditions, and hydrology (Treat et al. 2014; Saunois et al. 2020; Valentine et al. 1994; Wania et al. 2010; Whalen 2005). All of these are rapidly changing in the Arctic‐Boreal domain, which already accounts for some of the largest proportion of global wetlands, particularly peatlands containing a disproportionate amount of carbon stocks (Melton et al. 2013). Arctic‐Boreal wetland CH_4_ emissions are estimated to emit approximately 15–52 Tg CH_4_ annually (Ying et al. 2025). Emissions have shown increasing trends with warming and ecosystem productivity, which is associated with additional organic matter inputs (Yuan et al. 2024) as well as microbial activity (Oh et al. 2022). Yet, future and even current emissions remain uncertain due to (1) a lack of site‐specific long‐term, year‐round carbon flux measurements across the high‐latitudes (Pallandt et al. 2022; Euskirchen et al. 2017) with even fewer sites capturing CH_4_ fluxes across land cover classes (Kuhn et al. 2021), (2) uncertainty in mapping present and future wetland distribution and types (Olefeldt et al. 2016; Turetsky et al. 2020), and (3) very few models representing both permafrost and CH_4_ dynamics (Matthes et al. 2025).

An increasing number of process‐based terrestrial biosphere models (TBMs; Xu et al. 2016) are capable of predicting a substantial portion of the PCCF (Fisher et al. 2014) by representing the main processes driving interactions between climate, permafrost and carbon dynamics in soils, vegetation and the atmosphere (Tao et al. 2020; Zhuang et al. 2010; Genet et al. 2018; Briones et al. 2024; McGuire et al. 2016). However, recent model intercomparisons have revealed large uncertainties in estimations of the PCCF (McGuire et al. 2018; Huntzinger et al. 2020) with varied model sensitivities (McGuire et al. 2016). Most TBMs, the land surface component of Earth System Models (ESMs), are lacking appropriate representation of permafrost carbon (Schädel et al. 2024; Matthes et al. 2025). Among models able to represent the PCCF, few include the processes driving CH_4_ emissions from wetlands. Not all models predict methanogenesis, oxidation, and the main transport pathways (diffusion, ebullition, aerenchyma) and when they do, methods often differ (e.g., ebullition based on concentration rather than volume threshold; Ueyama et al. 2023; Tang et al. 2010). Additionally, few studies have examined the uncertainty associated with the fraction of CH_4_ emitted via each transport pathway, though this is challenging to measure and poorly constrained.

In this work, we investigate the sensitivity and uncertainty of Arctic‐Boreal CH_4_ emissions in the context of historical and future climate change, exploring the interplay between permafrost thaw, vegetation dynamics, and hydrology. We represented methanogenesis, oxidation, and the main transport pathways within a process‐based TBM—specifically developed for high latitude ecosystems—which represents the interplay between soil thermal and hydrological regimes, their interactions with vegetation and the carbon cycle. We calibrated and benchmarked our model against observations from a thermokarst collapse scar bog in interior Alaska, a site with active permafrost degradation (Manies et al. 2021). We then investigated the sensitivity and uncertainty of model predictions to biogeochemical and biophysical parameters, input data, and dominant CH_4_ emission transport pathways, using multiple downscaled climate projection scenarios.

## 2 Materials and Methods

To assess model sensitivity and uncertainty of CH_4_ emissions and radiative forcing in high latitudes, we integrated a CH_4_ flux model into a state‐of‐the‐art TBM. We used the dynamic vegetation model (DVM; Euskirchen et al. 2009) and dynamic organic soil (DOS; Yi et al. 2010) version of the Terrestrial Ecosystem Model (TEM; McGuire et al. 1992), DVM‐DOS‐TEM, a model developed specifically for high latitude ecosystems (Carman et al. 2025). Subsequently, we calibrated and benchmarked the model against observations of CH_4_ and CO_2_ fluxes, organic carbon stocks, and thermal and hydrological dynamics collected at a thermokarst bog located in interior Alaska. Parameters and input data were varied to evaluate correlations and long‐term model stability through a sensitivity experiment. The transport pathway dominance was varied by tuning specific parameters to determine the effects on CH_4_ seasonality and future projections. Finally, we made future predictions to the end of the century using downscaled Coupled Model Intercomparison Project phase 6 (CMIP6; Eyring et al. 2016) data with four Shared Socioeconomic Pathways (SSPs) and corresponding Representative Concentration Pathways (RCPs; SSP1‐2.6, SSP2‐4.5, SSP3‐7.0, SSP5‐8.5) from two ESMs (ACCESS and MRI).

### 2.1 Site Description

**FIGURE 1:**
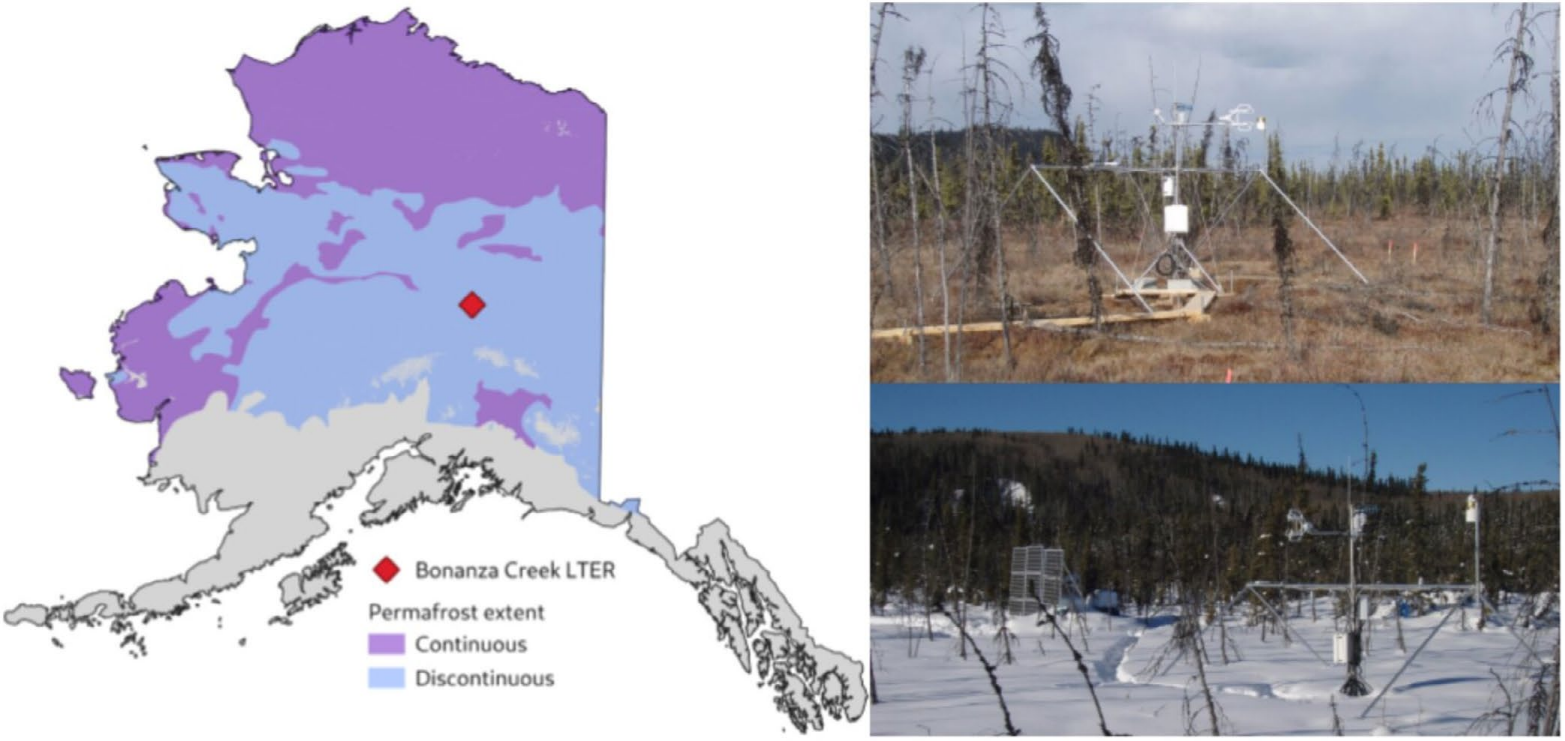
Location of Bonanza Creek Long term ecological research (LTER) site shown in Alaska over continuous and discontinuous permafrost extent (Heginbottom et al. 2002), flux tower and boardwalks located at the intermediate to old age thermokarst collapse scar bog site, part of the Alaska Peatland Experiment during summer and winter. Photo credit: Colin Edgar.

The site chosen for our simulations was a thermokarst collapse scar bog (Figure 1), estimated to have begun thawing 50–140 years ago based on macrofossil dating (Waldrop et al. 2021; Manies et al. 2021; referred to by the Ameriflux site name US‐BZB). Bog age has been shown to affect seasonal ice depth, gas emission, and carbon source (Klapstein et al. 2014), though hydrological dynamics are similar across ages. The site is located at (64.6955° N, 148.3208° W) within the Bonanza Creek Long‐term Ecological Research (LTER) Experimental Forest, in the Tanana Flats region of interior Alaska on discontinuous permafrost extent (Figure 1).

Mean annual air temperature (MAAT) was −0.8°C and precipitation averaged 279 mm between 2014 and 2022 according to subhourly meteorological data collected at the site. US‐BZB is surrounded by permafrost plateaus forested predominantly by black spruce ( *Picea mariana* ) trees. This site has both net ecosystem exchange (NEE) and CH_4_ flux data available through Ameriflux (Euskirchen 2025) with continuous biological and meteorological measurements over the study period from 2014 to 2022. Additionally, the site is part of the Alaska Peatland Experiment (APEX), which began in 2005 and has been studied extensively, providing ancillary data for model validation and benchmarking (e.g., Neumann, Moorberg, et al. 2019; Neumann, Mooreberg, et al. 2019; McConnell et al. 2013; James, Minsley, McFarland, et al. 2021; James, Minsley, Waldrop, et al. 2021; Churchill 2011). Collapse scar bogs and fens are prevalent in the Tanana Flats region, which is a hot spot for thermokarst activity, forming over hundreds of years as circular depressions with active thaw margins at the perimeters (Jorgenson et al. 2020). Bogs and permafrost bogs have been found to be the most abundant wetland classes across the Arctic‐Boreal domain (Olefeldt et al. 2021). Hence, this site offered representative dynamics which are relatable across wider regions.

### 2.2 Model Description

DVM‐DOS‐TEM is an open‐source, ecosystem model (Zhuang et al. 2001, 2002; Yi et al. 2009; McGuire et al. 2018; Carman et al. 2025). It is an offline model, meaning it does not include atmospheric feedbacks. Biogeochemical processes are captured by dynamically simulating carbon (C) and nitrogen (N) cycling between soil and vegetation (Raich et al. 1991; Tian et al. 1999). The model can be applied regionally in a spatially‐explicit manner (e.g., Genet et al. 2018), or at the site‐level (e.g., Briones et al. 2024). Vegetation is classified by community types (CMTs), defined as representative grouped vegetation and soil characteristics. Each CMT is composed of up to 10 plant functional types (PFTs), groups of species sharing similar ecological traits. C and N dynamics within each PFT are simulated for leaf, stem, and root compartments. PFTs compete dynamically for light, water and nutrient availability (Euskirchen et al. 2009). The soil column is composed of multiple layers (up to 22) with 4 horizon types (live moss, fibric and humic organics, and mineral) over a parent material (bedrock) that is used for imposing boundary conditions. Organic soil layer C stocks are calculated dynamically and converted to thicknesses, varying over time and impacting permafrost stability through changing insulative effects (Yi et al. 2010). C and N cycling can be modulated by climate and disturbances such as wildfire (Genet et al. 2013, 2018; Kelly et al. 2016). DVM‐DOS‐TEM has been applied to both permafrost and non‐permafrost ecosystems (Yi et al. 2009; Genet et al. 2013, 2018; Jafarov et al. 2013; Euskirchen et al. 2022; Briones et al. 2024). Biophysical processes (e.g., energy balance) are run on a pseudo daily time step (linear interpolation from monthly climate forcings), and biogeochemical processes are simulated on a monthly basis. DVM‐DOS‐TEM utilizes a spinup approach using averaged climate forcings to equilibrate biophysical and biogeochemical state variables prior to simulating the historic or projected, transient processes (Jafarov et al. 2025).

Soil thermal regimes are calculated by solving the heat equation, with layers parameterized with thermal conductivities depending on soil horizon type and modulated by porosity and the water and ice content in those layers (Zhuang et al. 2001, 2002). A two‐directional Stefan algorithm (Woo et al. 2004; Yi et al. 2009; Gao et al. 2016) is employed to calculate freezing/thawing fronts, which are used to determine thaw depth and thus a perched water table using the Richards equation (Dingman 2015).

Soil moisture is calculated by considering a one‐dimensional water balance, similar to the TOPMODEL with surface and subsurface runoff (Oleson et al. 2008; Swenson et al. 2012). Precipitation input forcings are first partitioned into rain and snow. Canopy interception, throughfall, and drip are calculated, followed by evaporation, surface runoff, and infiltration. Snow density is calculated and updated over time and under further precipitation, moderating thermal conductance into the ground layers, as well as sublimation. A ponding depth is assumed to store surface water if top soil layers become saturated, which will either infiltrate later or be lost as runoff. Percolation through soil layers is then calculated with subsurface drainage in the lowest unfrozen layer included depending on whether a cell is assigned well‐ or poor‐draining conditions.

For lowlands and wetlands with a poor‐draining assignment, runoff losses are assumed zero and a run‐on component is added to account for overland flow entering the cell from surrounding microtopography. An approximation for ground water recharge, upwelling, or subsurface inputs through lateral drainage channels is estimated following a formulation similar to subsurface drainage (Oleson et al. 2008; Swenson et al. 2012) using a calibrated scalar opposed to soil moisture content. This is proportional to the cell slope, hydraulic conductivities and thicknesses of the layers. This approach is used to improve model representation of soil hydrological regimes, where indices to inform approximate groundwater flow have been shown to improve predictions of soil moisture (Rodhe and Seibert 1999; Meles et al. 2020; Persson et al. 2012; Sato et al. 2020; Kåresdotter et al. 2021).

### 2.3 CH_4_ Flux Model Integration

**FIGURE 2:**
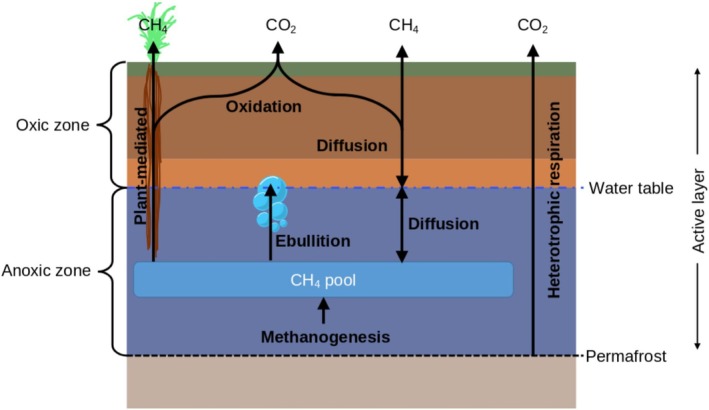
Conceptual schematic diagram of CH_4_ flux calculations integrated into DVM‐DOS‐TEM. Representing delineation of oxic and anoxic zones via the water table perched above the permafrost within the active layer. Anoxic processes include methanogenesis and ebullition. Oxic processes include CH_4_ oxidation, with diffusion taking place throughout the soil column modified based on corresponding diffusion and tortuosity coefficients. Aerenchyma‐transport takes place throughout the rooting depth independent of water table position, but also contributes to oxidation. Heterotrophic respiration is calculated throughout the soil column and modulated by soil moisture in DVM‐DOS‐TEM prior to CH_4_ flux integration.

Integration of CH_4_ flux calculations in DVM‐DOS‐TEM builds on the frameworks developed by (Zhuang et al. 2004) and (Fan et al. 2013), with processes shown schematically in Figure 2. A full description of the equations implemented in the model and a list of scientific notation used is provided in the Supporting Information. Determination of CH_4_ flux dynamics in permafrost ecosystems requires accurate simulation of soil temperature and moisture (Lawrence et al. 2015), in particular the effect of these variables on the seasonal position of the water table (the depth at which soil saturation drops below 90%). The water table is used to delineate soil layers as either oxic or anoxic. When a soil layer contains the water table, an interpolation approach is used to determine the ratio of oxic to anoxic processes to be calculated. Anoxic processes include methanogenesis and ebullition, while oxidation is the primary process in oxic layers. Plant‐mediated (aerenchyma; enabled through the spongy tissues of certain plants such as *Carex*) transport of CH_4_ is calculated within the rooting zone and diffusion throughout the soil column, and parameters are modified depending on whether the layer is saturated or unsaturated.

Methanogenesis is calculated in each layer below or containing the water table as the product of a calibrated rate limiting constant, a Q10‐based temperature sensitivity function, and the C substrate availability in each soil organic matter pool (raw litter, active, physically bound, and chemically resistant). Methanogenesis feeds a CH_4_ pool that is updated for each layer at every time step. CH_4_ oxidation is calculated in each layer above or containing the water table as the product of the concentration of CH_4_ in the corresponding layer pool, another Q10‐based temperature sensitivity function, and a Michaelis–Menten kinetics function similarly to Walter and Heimann (2000). Two Michaelis–Menten kinetics parameters are used during the calibration process to balance oxidation and moderate the level of emission via diffusion.

Aerenchyma transport is calculated from the fine root fraction in each soil layer and is dependent on the rooting depth rather than the water table position. Approximately 50% of the CH_4_ emitted through aerenchyma is assumed to be oxidized in the rhizosphere, as in Walter and Heimann (2000). Though it has been shown in certain cases that this may be an overestimate (Turner et al. 2020), we maintain 50% due to a lack of observational data to parameterize this process. In terrestrial ecosystems, aerenchyma transport is dominant (Laanbroek 2010; Schimel 1995; Van Der Nat and Middelburg 1998) and will decrease overall oxidation through bypassing the aerobic soil layers (Marushchak et al. 2016; Knoblauch et al. 2015). Molecular diffusion is calculated through the entire soil column using diffusion and tortuosity constants based on layer saturation. The model is initialized at time zero for spinup with atmospheric CH_4_ concentration in each soil layer and then the emissions are spun up to equilibrate CH_4_ processes before representing the historical time period.

Layers are looped from lower (~6 m depth) to the upper boundary of the soil column, with ebullitive transport calculated first. This allows CH_4_ above a temperature‐dependent concentration threshold within a given layer to be redistributed to the layer containing the water table (provided the layer is not completely frozen) before calculation of individual layer processes requiring updated CH_4_ concentration. This assumption is based on measurements of the velocity of bubbles in water on the order of 1–10 cm/s and the porous wetland and peatland soils common in high latitudes (Walter and Heimann 2000), suggesting ebullitive flux occurs below the time scale of other transport processes. A single‐substance (tracking only a single gas, e.g., CH_4_), temperature‐dependent threshold function is used to calculate the CH_4_ concentration above which gas is released through ebullition. Though Ma et al. (2022) demonstrated enhanced prediction utilizing a volume‐based ebullition threshold, it has been shown that annual CH_4_ predictions do not vary between ebullition models, with temporal variation observed at daily and sub‐daily timescales (Peltola et al. 2018). As DVM‐DOS‐TEM is designed for large‐area (e.g., circumpolar domain) simulations we utilize the concentration threshold approach, similarly to Walter and Heimann (2000), Zhuang et al. (2004), Wania et al. (2010), Fan et al. (2013), and others. Ma et al. (2022) report the need for combined CH_4_ flux and concentration data to better constrain parameters in ebullition model approaches. The volume‐threshold approach is recommended in modeling efforts with higher temporal resolution input forcings or provided there are sufficient observations to partition CH_4_ emission pathways.

When the water table is not at the soil surface, an exponential function is used to calculate a fraction of this ebullitive flux to leave the soil column depending on the depth and the porosity of the layer. This allows control over the episodic emissions across larger cell scales (> 1 km^2^), representing variability in water table depth and heterogeneity in soil surface microtopography (with hummocks and depressions common in peatlands). Observations show that bubbles move at high speeds through porous soils such as peat (Scheffer and Schachtschabel 1982; Shafer and Zare 1991) and flux occurs in the upper 90% of peat layers (Klapstein et al. 2014), though there is associated heterogeneity (Korrensalo et al. 2018).

Molecular diffusion is calculated following determination of other flux components (methanogenesis, oxidation, aerenchyma transport, and ebullition). The reaction–diffusion equation is solved using a Crank–Nicolson discretization (Crank and Nicolson 1947) with the tridiagonal matrix algorithm (Thomas 2013). The diffusion coefficient of each layer is calculated from the diffusion constant of CH_4_ in either air or water and the tortuosity of the soil (describing the movement of gas through pores in the soil), which is scaled based on the temperature of the layer. Finally, we assume open (Dirichlet) and closed (Neumann) boundary conditions at the top and bottom of the soil column, respectively. This represents diffusion into (uptake) or out of (efflux) the top layer of the soil column relative to an atmospheric CH_4_ concentration input forcing, and no transmission of CH_4_ into the bed rock below the soils.

### 2.4 Model Calibration

DVM‐DOS‐TEM employs a calibration process to tune rate‐limiting parameters for biogeochemical processes. This allows for improved site representation and can help when simulating spatially through the implementation of precise land cover classes (Briones et al. 2025). The process involves conducting ensemble runs with parameters sampled between predefined literature ranges where available (see Table S1), following a process similar to (Briones et al. 2024; Jafarov et al. 2025). Averaged pre‐industrial climate is used to reflect observational data for C and N fluxes, biomass, structural N, and soil C stocks under stable state conditions. Ensembles are run until equilibrium is reached (~200 years for vegetation and ~2000 years for soils). We added calibration of CH_4_‐specific parameters, including rate limiting constants, methanogenesis and oxidation Q10 and reference temperatures, and Michaelis–Menten parameters *k*_m_ and *V*_max_. Observations of vegetation C and net primary productivity (NPP) for the calibration process were derived from the Bonanza Creek LTER Data Catalog (Churchill 2011, 2014a, 2014b, 2014c, 2014d, 2014e). Vegetation N, C:N, and N uptake, were estimated from studies at the site or sites of similar characteristics (Finger 2015; Wieder et al. 2020; Nordbakken et al. 2003; Li and Vitt 1997). Soil core data, including characterization of soil C, N, bulk density, and horizon depths were collected by (Manies et al. 2017). CO_2_ and CH_4_ fluxes from Ameriflux BASE (Euskirchen 2025) were used as these provided the longest CH_4_ time series.

### 2.5 Benchmarking, Sensitivity and Uncertainty Analysis

We simulated the historical period with the parameterization developed during calibration using downscaled CRU‐JRA TS v.4.0 (Harris et al. 2020) climate input data to compare model outputs to observational data (Euskirchen 2025; James, Minsley, McFarland, et al. 2021; James, Minsley, Waldrop, et al. 2021; Neumann, Moorberg, et al. 2019; Neumann, Mooreberg, et al. 2019).

We conducted three sensitivity assessments: (1) ensemble simulations with perturbation of biogeochemical and biophysical parameters, (2) modification to summer and winter air temperature and precipitation input forcing data, and (3) variation in dominant CH_4_ efflux transport pathway. We assessed future projections and model uncertainty by simulating CO_2_ and CH_4_ fluxes and radiative forcing using downscaled CMIP6 climate data (Eyring et al. 2016) with four scenarios: SSP1‐2.6, SSP2‐4.5, SSP3‐7.0, and SSP5‐8.5 from two ESMs: ACCESS and MRI.

We used an ensemble model approach with a sample size of 500, varying biogeochemical and biophysical parameters separately within literature‐derived ranges where available (see Table S1) using a latin‐hypercube sampling method to cover the intended parameter space (Briones et al. 2024). Heterotrophic respiration and methanogenesis rate‐limiting constants were ranged by ±50% of the calibrated value, as these are highly sensitive but uncertain variables (Euskirchen et al. 2009). Samples were run for 1000 years with outliers removed if stable state conditions were not reached. Correlations between parameters and output variables were analyzed to determine direction and magnitude. We evaluated long‐term stability and sensitivity by modifying input data and analyzing the change in model outputs when run under averaged climate. Variation in simulated outputs was evaluated by modifying summer and winter air temperature and precipitation by ±1°C, 2°C, and 3°C and 5, 10, and 15 mm (Figure S3) respectively (Yi et al. 2009). Air temperature and precipitation were modified independently and sequentially. We then calculated a resulting sensitivity index (Equation 9; Friend et al. 1993). Sensitivity (*S*) is calculated from the effective change between altered (*a*) and baseline (*bl*) predicted variable (*v*) and input (*I*) ratios, quantifying the sensitivity of the predicted value to changes in input (e.g., the sensitivity of CH_4_ emissions to summer air temperature).

(9) $$S=\frac{v_{a}-v_{\mathrm{bl}}}{v_{\mathrm{bl}}}/\frac{I_{a}-I_{\mathrm{bl}}}{I_{\mathrm{bl}}}$$

We assessed the variations associated with CH_4_ transport pathway equifinality (multiple combinations of parameters resulting in similar flux predictions; Ma et al. 2022).

Once heterotrophic respiration and methanogenesis parameters were calibrated against observations of soil C stocks and ecosystem respiration (RECO), parameters controlling transport pathway dominance were varied. These were rate limiting parameters associated with CH_4_ efflux via aerenchyma and ebullition. Michaelis–Menten kinetics parameters were varied to modify oxidation and thus CH_4_ efflux via diffusion. This maintained soil C stocks while changing the dominance of the emission transport pathway (i.e., > 50% ebullition). Parameters were varied to best match mean annual CH_4_ observations while shifting pathway dominance (accounting for the largest ratio of CH_4_ efflux). After parameterizations were derived, we assessed differences in seasonality and the uncertainty with future climate projections. Predictions to the end of the century were made across four SSPs and two ESMs. Projected decadal air temperature and precipitation for each ESM and all scenarios are shown in Figure S6. ACCESS and MRI represented warmer and drier, and cooler and wetter projections respectively. We used model projections to evaluate the uncertainty our parameterization may have on the C budget. From predicted CO_2_ and CH_4_ fluxes we calculated radiative forcing using Equation (10) following Frolking and Roulet (2007). This method allowed us to integrate predicted time series of CO_2_ and CH_4_ fluxes to calculate net radiative forcing for the site. RF_total_ is the radiative forcing at time t as a sum of the individual gas contributions (where CO_2_ is composed of five indices 0–4 and CH_4_ one index 5 to represent reservoirs of the gas species with a spread of residence times). *ξ* is a multiplier for indirect effects (1.0 and 1.3 for CO_2_ and CH_4_ respectively). *A*_*i*_ is the radiative efficiency of the gas index *i* (0.0198 × 10^−13^ Wm^−2^ kg^−1^ and 1.30 × 10^−13^Wm^−2^ kg^−1^ for CO_2_ and CH_4_ respectively). *f*_*i*_ is a fractional multiplier per index (with values 0.176, 0.138, 0.186, 0.242, and 0.259 for CO_2_ indices and 1.0 for CH_4_). *Ф*_*i*_(*t*′) is the net flux of gas index into the atmosphere at time *t*′. *τ*_*i*_ is an adjusted residence time. More details are provided in Frolking and Roulet (2007).

(10) $$RF_{\text{total}}\left(t\right)=\sum_{i=0}^{5}\left(\xi_{i}A_{i}f_{i}\int_{0}^{t}\Phi_{i}\left(t^{\prime }\right)e^{\left(t^{\prime }-t\right)/\tau_{i}}{\mathrm{dt}}^{\prime }\right)$$

Projections were repeated using our calibrated parameterization (which was dominated by aerenchyma CH_4_ emissions) and parameterizations where ebullition and diffusion were set as the dominant CH_4_ emission pathway to evaluate the uncertainty associated with CH_4_ emission transport pathway dominance.

## 3 Results

We report simulated outputs for calibration, benchmarking, sensitivity, and uncertainty analyses, comparing where possible to observational data associated with, or representative of, the flux tower US‐BZB (Euskirchen 2025; James, Minsley, McFarland, et al. 2021; James, Minsley, Waldrop, et al. 2021; Neumann, Moorberg, et al. 2019; Neumann, Mooreberg, et al. 2019).

### 3.1 Calibration

The results of calibration for gross primary productivity (GPP), vegetation C, soil organic C in fibric, humic, and mineral (top 1 m) horizon stocks, RECO, and CH_4_ efflux were recorded in Table 1. Values are given annually, as sums and means for fluxes and stocks respectively. Humic C was most overestimated by 35%, though total soil organic C is 4% lower than observed. CH_4_ efflux was calibrated under climate conditions of our study period to better match seasonality when adjusting transport pathway‐related parameters.

**TABLE 1:** Observed and simulated state C flux and stock variables under steady state conditions (averaged climate 1901–1930) for 1000 years.

State variable (units)	Observed	Simulated
GPP (g C m^−2^ year^−1^)	433 ± 42 (Euskirchen 2025)	525
Vegetation C (g C m^−2^)	454 (Churchill and Bonanza Creek LTER 2014b, 2014e)	576
Fibric C (g C m^−2^)	8732 (Manies et al. 2017)	9324
Humic C (g C m^−2^)	24,092 (Manies et al. 2017)	32,524
Mineral C (g C m^−2^)	61,545 (Manies et al. 2017)	48,589
RECO (g C m^−2^ year^−1^)	374 ± 22 (Euskirchen 2025)	358
CH_4_ efflux (g C m^−2^ year^−1^)	11.07 ± 0.85 (Euskirchen 2025)	9.76^a^

*Note:* Standard deviation is shown with carbon fluxes as a measure of interannual variability.

^a^ CH_4_ was calibrated under climate conditions of our study period due to sensitivity and necessity for consideration of seasonal dynamics.

### 3.2 Benchmarking

**FIGURE 3:**
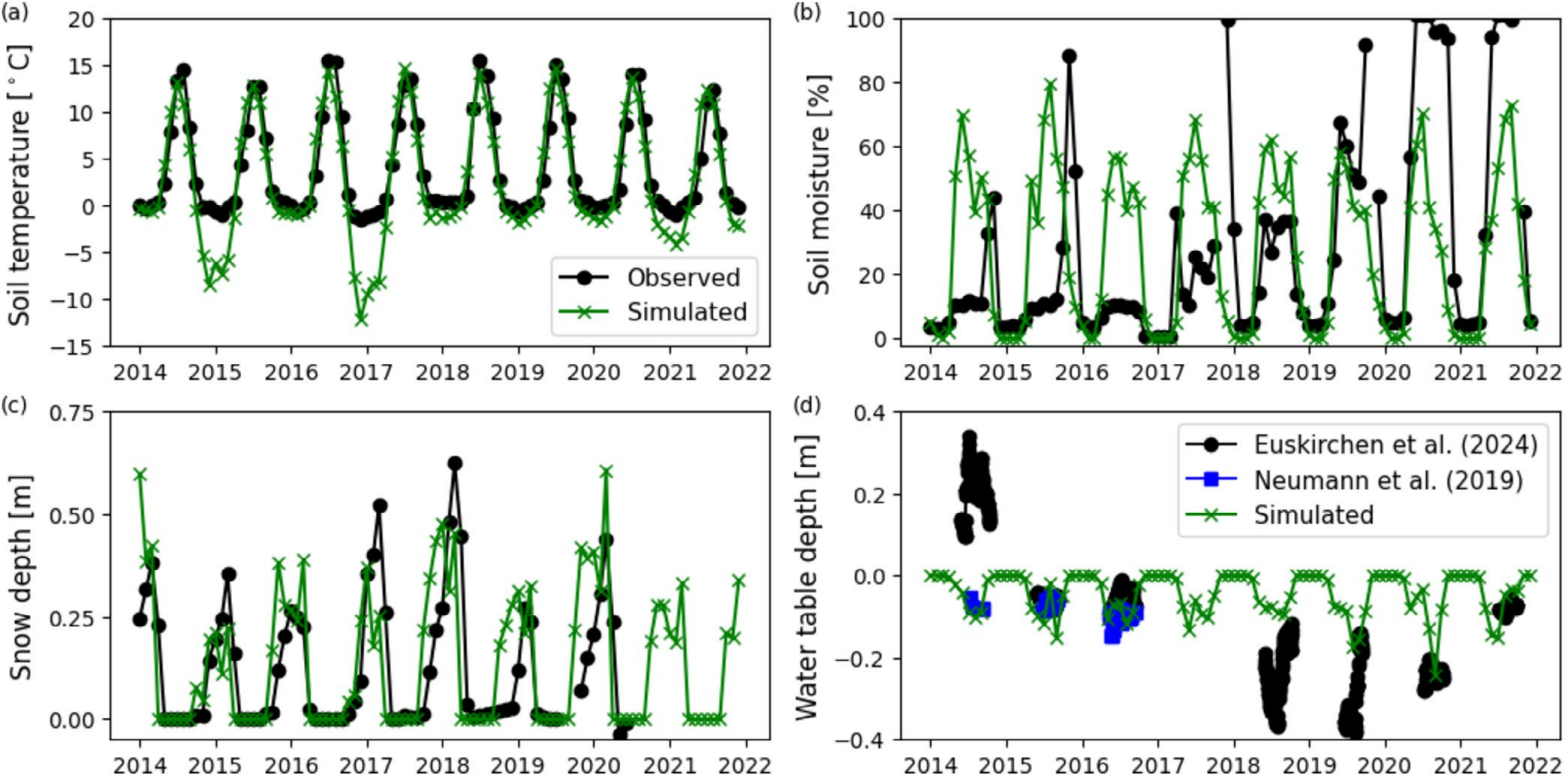
Observed and simulated monthly (a) soil temperature at 6 cm depth, (b) soil moisture at 6 cm depth, (c) snow depth, and (d) water table depth (Euskirchen et al. 2024; Neumann, Moorberg, et al. 2019; Neumann, Mooreberg, et al. 2019) across the observation period 2014–2022. For water table depth, positive values indicate inundation above the soil surface.

Soil temperature (Figure 3a) was adequately represented, though winter temperatures were underestimated between years 2014–2015 and 2016–2017, likely due to underpredicted inundation in the summer or snow depth in the winter (Figure 3c), allowing for deeper penetration of cold winter temperatures. Simulated active layer depth was ~1 m, which was within the observed ranges, though measurements were not taken at the center of the bog where thaw is deeper (James, Minsley, McFarland, et al. 2021; James, Minsley, Waldrop, et al. 2021). Soil moisture (Figure 3b) was overestimated prior to 2019 and underestimated after. Simulated summer water table depth matched both sets of observations between 2015 and 2017.

**FIGURE 4:**
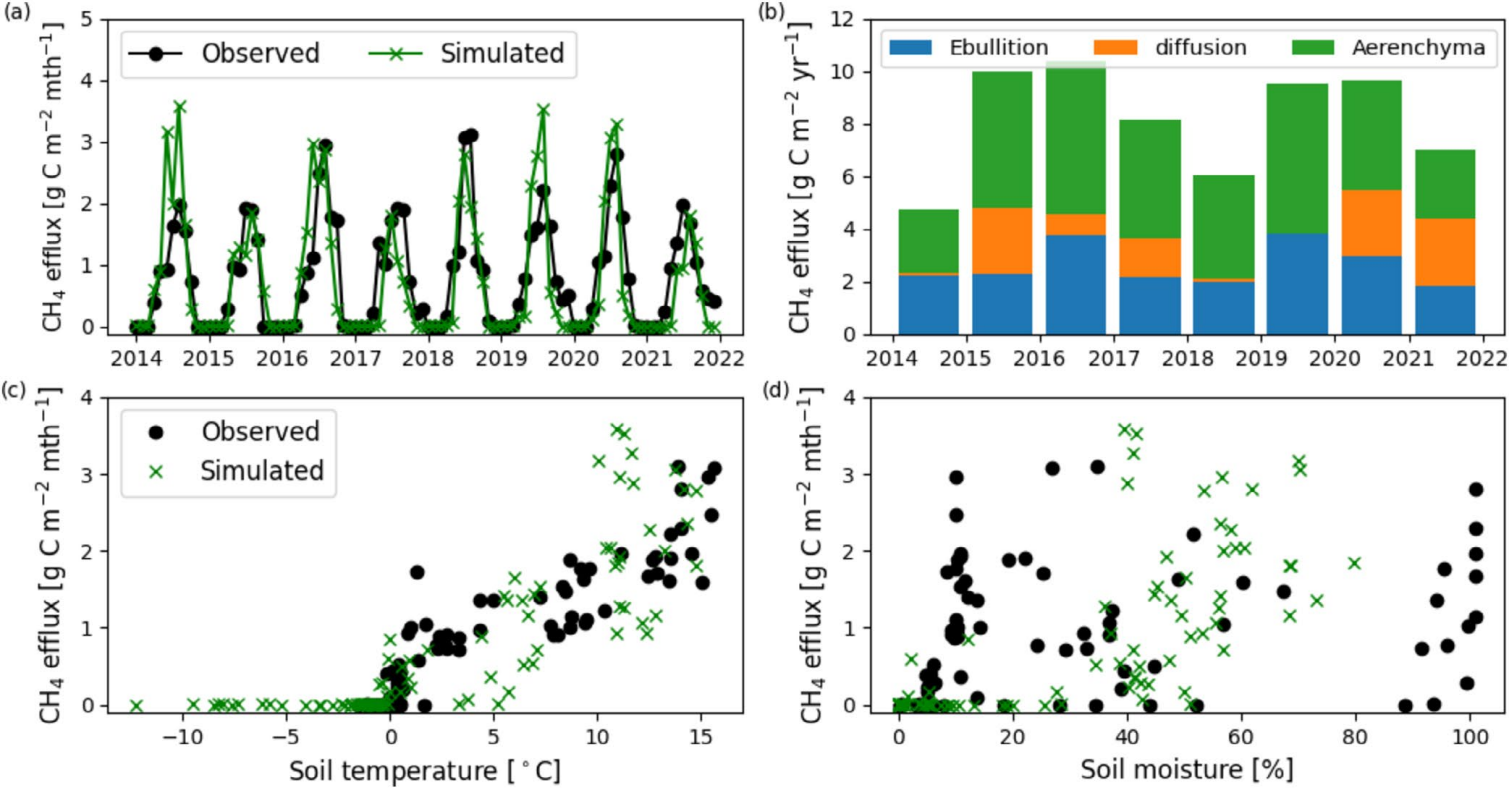
Observed and simulated (a) monthly total CH_4_ efflux, with (b) annual CH_4_ efflux contributions from ebullition, diffusion, and aerenchyma transport related efflux. Observed and simulated monthly CH_4_ efflux response against (c) soil temperature, and (d) soil moisture at 6 cm depth across the observation period 2014–2022.

Total CH_4_ efflux and partitioning by transport pathway were compared to time series observations of total CH_4_ in Figure 4a. The final calibrated parameterization was dominated by aerenchyma CH_4_ efflux, which will be referred to as the aerenchyma‐dominant parameterization (the largest fraction of CH_4_ emissions from aerenchyma transport pathway due to the combination of rate limiting constants found to capture seasonal observations).

The annual mean and range of seasonal and cumulative monthly CH_4_ efflux is shown in Figure 6 (which also shows deviations with transport pathway dominance). Shoulder seasons showed discrepancies from observations, particularly following summer due to early decline in simulated aerenchyma CH_4_ efflux. Peak season and mean cumulative CH_4_ efflux closely matched observations. The response of CH_4_ efflux compared to both soil temperature and volumetric soil moisture at 6 cm depth is shown in Figure 4c,d. Each point represents a monthly model output or observation. The simulated response of CH_4_ efflux to soil temperature at 6 cm depth matched the observed relationship for temperatures above freezing, though there was a discrepancy due to underpredicted soil temperatures between years 2014–2015 and 2016–2017 likely because of hydrological underpredictions. Both simulated and observed responses of CH_4_ efflux to soil moisture at 6 cm depth showed weak relationships. Simulated soil moisture was not > 80%, whereas observations reached 100% in the last 2 years of study and may be due to dynamics not accounted for in the model such as lateral flow and advective heat transport (Neumann, Moorberg, et al. 2019; Neumann, Mooreberg, et al. 2019).

### 3.3 Sensitivity Analysis

**FIGURE 5:**
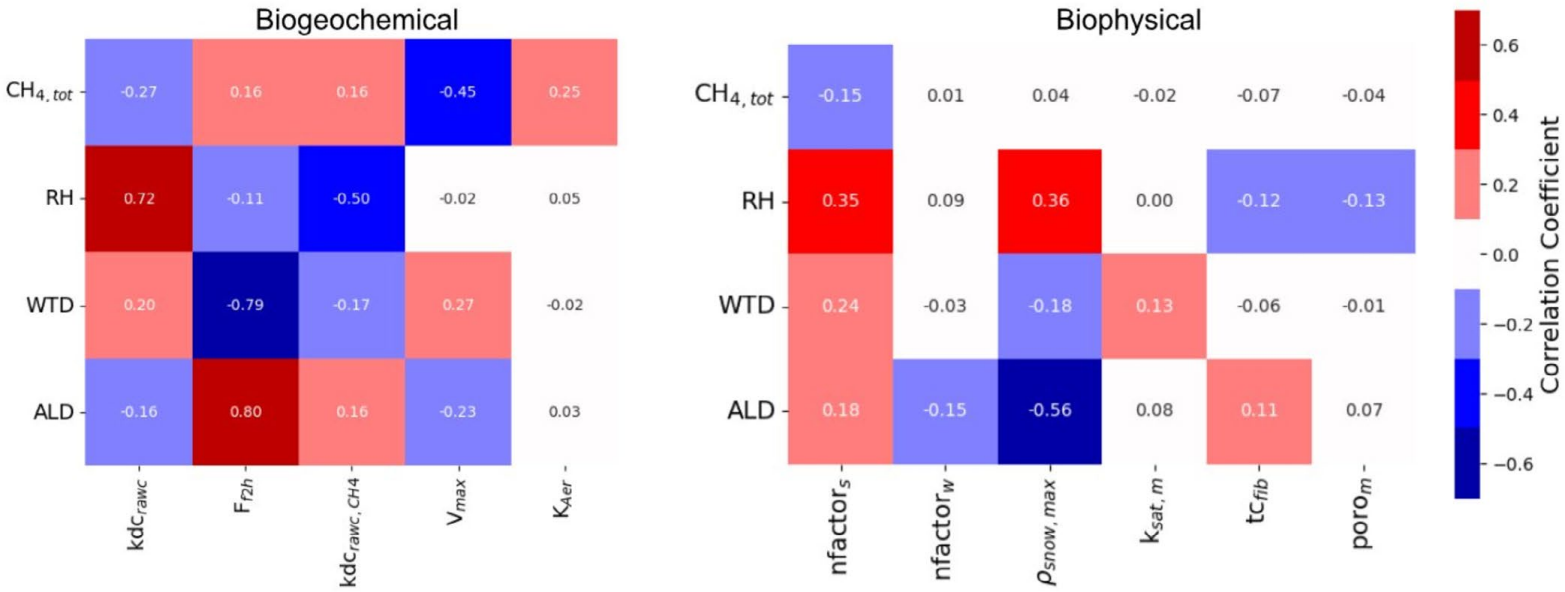
Matrices showing correlation between output variables, biogeochemical, and biophysical parameters.

Correlations between select model output variables, biogeochemical and biophysical parameters are shown in Figure 5. A full list of correlations between biogeochemical and biophysical parameters and model output variables are shown in Figures S1 and S2. We output CH_4_ efflux as a total (CH_4,Tot_) and emitted via aerenchyma (CH_4,Aer_), ebullition (CH_4,Ebul_), diffusion (CH_4,Diff_), methanogenesis (CH_4,Prod_) and oxidation (CH_4,Oxid_). Additional variables include heterotrophic respiration (RH), soil C by horizon (fibric, C_Fib_; humic, C_Hum_; Mineral, C_Min_), water table depth (WTD), and active layer depth (ALD). Parameters in Table S1 were varied, but only those reported had a correlation > 0.1 for at least one output variable. Figure 5 shows CH_4,Tot_, RH, WTD, and ALD as these variables captured the dynamics of the model. CH_4_ and RH show inverse correlations with their corresponding rate limiting parameters for the raw litter C pool (kdc_rawc_ and kdc_rawc,CH4_). The fibric to humic organic soil C burial factor (F_f2h_) showed a positive correlation with CH_4_ suggesting C substrate availability but also its distribution within the soil column are important drivers of CH_4_ emissions. The Michaelis–Menten kinetics parameter (*V*_max_) was negatively correlated with CH_4_ emissions through increased oxidation activity. The aerenchyma transport rate limiting parameter (K_Aer_) was also correlated with CH_4_ as our parameterization was aerenchyma‐dominant. WTD and ALD are driven by changes in summer and winter n‐factors (*n*factor_s_, *n*factor_w_) and maximum snow density (*ρ*_Snow,max_), which feedback on CH_4_ and RH. Hydraulic conductivity and porosity of moss (*k*_sat,m_ and poro_m_) and thermal conductivity of fibric organic soil (tc_fib_) also feedback primarily on RH, WTD, and ALD.

The normalized sensitivity index from input forcing data modification is reported in Figure S4. Data are normalized to the maximum response to air temperature and precipitation in respective columns. The relative response to input modification is shown in Figure S5 normalized to the maximum response to each input change (summer or winter, air temperature or precipitation), with the maximum sensitivity index shown on the figure. Additional variables, vegetation C (VEGC) and evapotranspiration (EET) are included. Results are calculated from the 10‐year mean following 1000 years of simulation under averaged climate conditions shown in Figure S3. These provide context to long‐term model stability following changes in climatic regime at this site.

**FIGURE 6:**
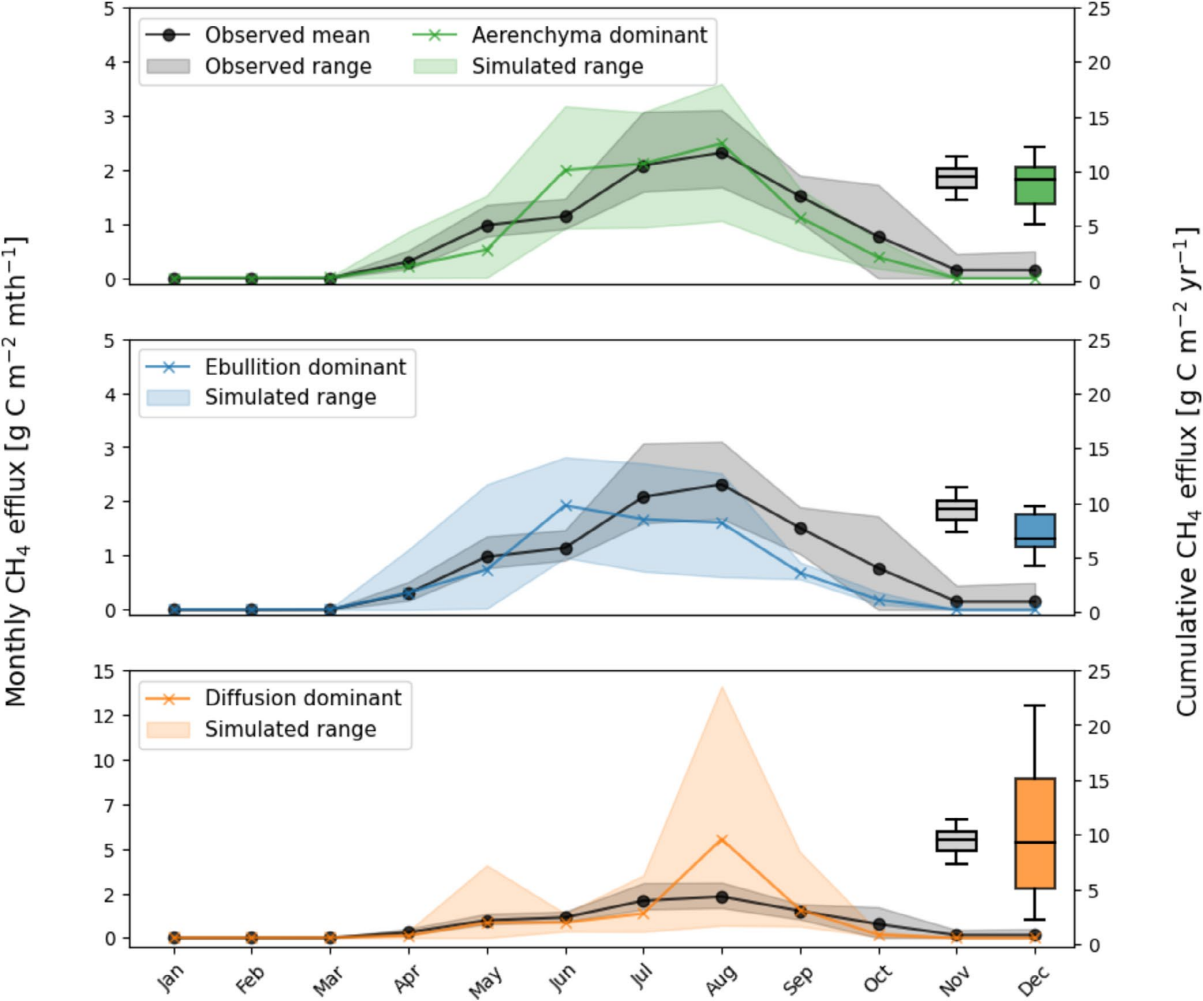
Simulated and observed monthly CH_4_ efflux with parameterizations forcing different dominant emission pathways aerenchyma‐dominant (final site calibration), ebullition‐dominant, and diffusion‐dominant. Solid lines show the mean and shaded areas the range between 2014 and 2022. Observations and simulations are shown with circular and cross markers, respectively. Box plots represent the variation in observed and simulated cumulative annual CH_4_ efflux.

We report the results of varying transport pathway dominance on simulated CH_4_ efflux (Figure 6). The simulated median cumulative CH_4_ efflux was within the range of observed values (shown by boxplots) for each transport pathway, though ebullition‐dominant was lowest and consistently underpredicted late season efflux. Diffusion‐dominant had the highest interannual variability, with overestimates in the shoulder seasons. Aerenchyma‐dominant (which was our final calibration) performed best in terms of capturing mean annual efflux, seasonality, and interannual variability. Soil C stocks were identically 89,635 g C m^−2^ at the beginning of the historical simulation period (1901‐01‐01) for aerenchyma, diffusion, and ebullition‐dominant transport pathway parameterizations, respectively. Both heterotrophic respiration and methanogenesis rate constants were unchanged, maintaining control for this sensitivity experiment.

### 3.4 Uncertainty Analysis

**FIGURE 7:**
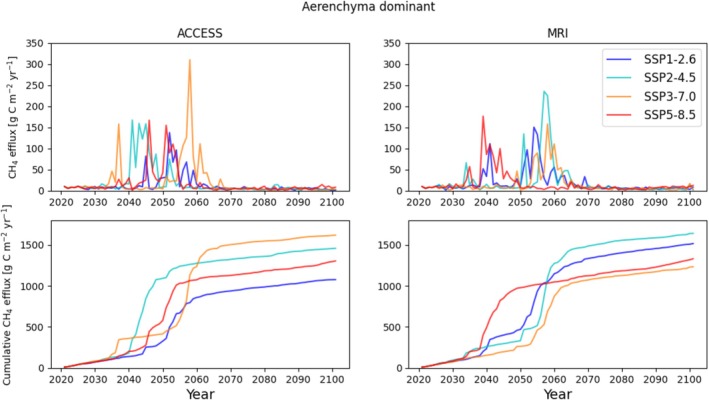
Projected annual CH_4_ efflux and cumulative annual CH_4_ efflux under ACCESS and MRI ESMs and SSP1‐2.6, SSP2‐4.5, SSP3‐7.0, SSP5‐8.5 using calibrated (aerenchyma‐dominant) parameterization.

We show projected annual and cumulative annual CH_4_ efflux under SSP1‐2.6, SSP2‐4.5, SSP3‐7.0, SSP5‐8.5 using ESMs ACCESS and MRI in Figure 7 with peak CH_4_ efflux showing a transient response. Cumulative CH_4_ efflux under SSP3‐7.0 and SSP2‐4.5 demonstrated the largest CH_4_ emissions by the end of the century for ACCESS and MRI, respectively. Projections of annual and cumulative annual CH_4_ efflux using ebullition‐ and diffusion‐dominant parameterizations are reported in Figures S7 and S8, with a similar transient behavior but with differing scenarios leading to the largest cumulative emissions. The ebullition‐dominant parameterization shows the largest discrepancy from our calibrated (aerenchyma‐dominant) parameterization. The diffusion‐dominant parameterization under SSP1‐2.6 for the MRI ESM produced the highest cumulative CH_4_ efflux by the end of the century. Projections of ALD and WTD showing the mean and range between scenarios, ESMs, and transport pathway dominance are shown in Figures S9 and S10, which explained the transient response of CH_4_ efflux, with more severe warming scenarios causing accelerated thaw and lowering of the water table (soil drying) preventing prolonged methanogenesis. Projections of RECO, GPP and NEE are reported by ESM and compared with transport pathway dominance in Figures S11–, S13. Projections of CH_4_ efflux showed a transient behavior increasing during the middle of the century but with a lag between SSPs and ESMs. The timing of peak CH_4_ efflux differed between SSPs and ESMs due to differences in temperature and moisture inputs. Peak efflux aligned with the onset of ALD increase but before WTD decreases, which resulted in drying of the top soil layers and reducing cumulative efflux by the end of the century. SSP3‐7.0 and SSP2‐4.5 produced the highest cumulative efflux by the end of the century for ACCESS and MRI respectively, whereas SSP5‐8.5 caused rapid thaw and lowering of the water table too quickly for prolonged methanogenesis.

**FIGURE 8:**
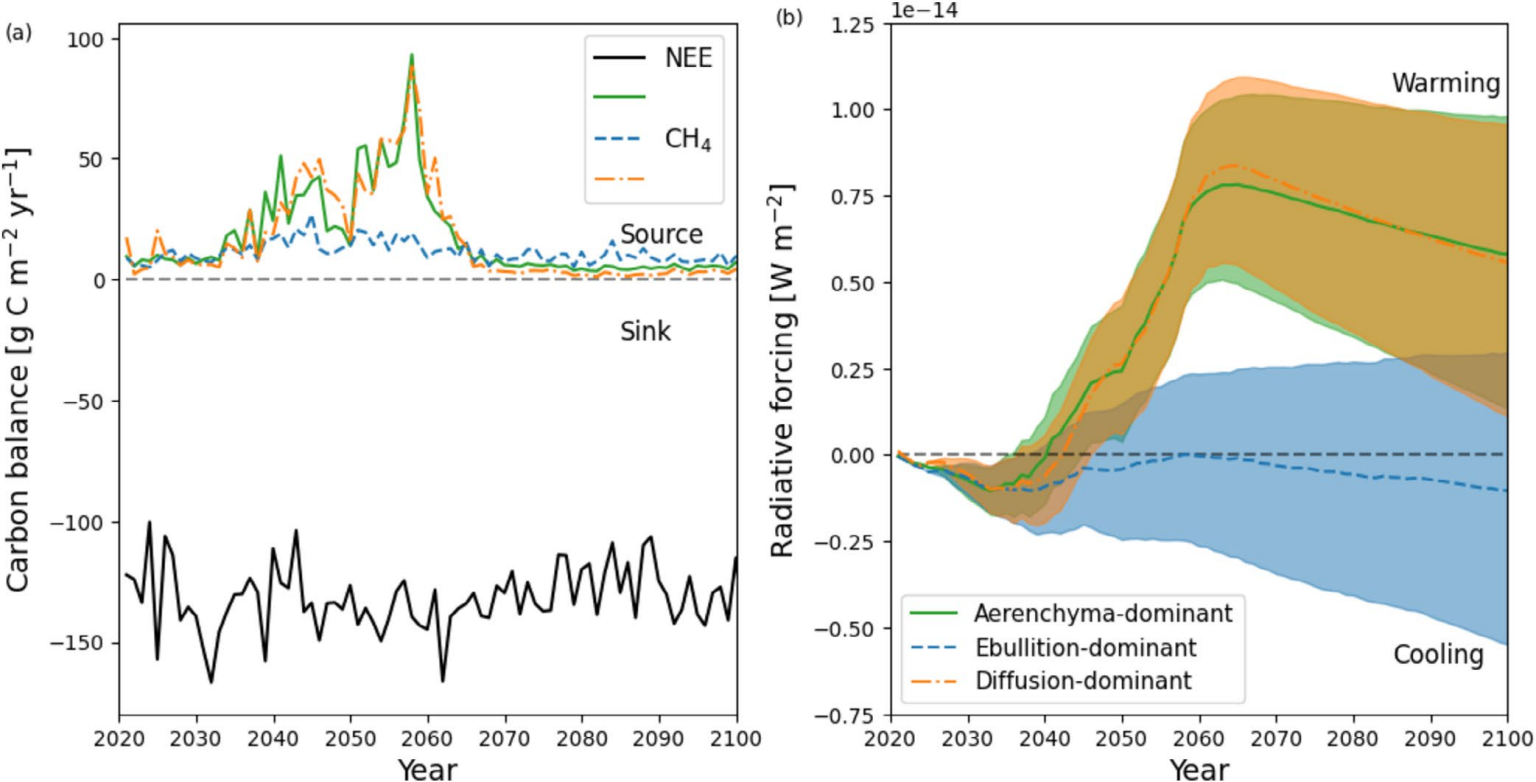
Projected (a) carbon balance partitioned by NEE and CH_4_, and (b) radiative forcing showing the mean and range of the projections from the ACCESS and MRI ESMs and SSP1‐2.6, SSP2‐4.5, SSP3‐7.0, SSP5‐8.5 for model parameterizations dominated by various transport pathways (aerenchyma, ebullition, diffusion). NEE remains constant between different transport pathway parameterizations.

Projected NEE and CH_4_ efflux (delineated by transport pathway dominance), with corresponding net radiative forcing, is shown in Figure 8a,b respectively. We report the mean and range between SSPs and ESMs for radiative forcing. NEE alone remained a C sink through the projected period, though the mid‐century CH_4_ emissions created a C source for a number of years. Though the C budget was weighted towards CO_2_‐related C uptake, when accounting for warming potentials and residence times of CH_4_ and CO_2_, we calculated a disproportionate positive radiative forcing starting in 2035 and persisting until the end of the century. Peak positive radiative forcing occurred around 2065 then steadily declined.

An uncertainty of 8.3 × 10^−15^W m^−2^ in the radiative forcing was predicted across SSPs and ESMs, though this remained consistently positive. Figure 8b shows the radiative forcing predicted across SSPs and ESMs for different transport pathway dominant model parameterizations. Aerenchyma‐ and diffusion‐dominant parameterizations predicted warming until the end of the century, whereas mean ebullition‐dominance predicted cooling.

## 4 Discussion

The calibration approach developed in this study suggested the need for long‐term soil CH_4_ concentration profiles, which could be used to better constrain subsurface dynamics. There are few long‐term datasets reporting soil gas concentrations at varying depths (James, Minsley, McFarland, et al. 2021; James, Minsley, Waldrop, et al. 2021), though these can assist in the calibration of methanogenesis parameters and could improve simulated C stock distributions by soil horizon (Table 1). Little information is available on CH_4_ emissions partitioning between transport pathways, greatly limiting our parameterization effort. However, the relative effect of the emission pathways on the seasonal patterns of total emissions assisted in our calibration process. The importance of considering seasonality in our calibration was reflected when we were unable to capture observed full season CH_4_ efflux without a balance of oxidation and transport pathways. Our final calibration was aerenchyma‐dominated, which is appropriate for this ecosystem (Turner et al. 2020). Nevertheless, ebullitive and diffusive efflux were required to bolster predictions of shoulder season efflux. This highlights the importance of considering seasonality when calibrating TBMs, particularly for CH_4_ dynamics.

DVM‐DOS‐TEM captured many of the thermal and hydrological dynamics at this site, though winter temperatures were underpredicted. Comparison with WTD and snow depth suggested this was due in part to inconsistent predictions of summer or winter hydrology, though additional processes (e.g., advective heat transport) are not captured in the model, which is important in lowlands (Neumann, Moorberg, et al. 2019; Neumann, Mooreberg, et al. 2019). Overpredictions of summer soil temperature and the rate of change in the shoulder seasons, and underpredictions in the winter may be improved with higher resolution temporal (e.g., hourly) climate input data and consideration of unfrozen water content (Maglio et al. 2024; Nicolsky and Romanovsky 2018). Soil moisture was overpredicted in years before 2019 and overpredicted after, however, the predicted WTD (used to delineate oxic and anoxic soil layers) largely captured summer dynamics. Inundation above the soil surface is also not represented in the model. As soil moisture data were collected at only 2 and 6 cm depth, this does not provide extensive information on the hydrological regime, as upper moss and organic soil layers tend to dry out in boreal bogs, but remain saturated in deeper layers (Harden et al. 1997). Inundated bogs are often characterized by a floating peat mat making accurate measurement with depth challenging (Neumann, Moorberg, et al. 2019; Neumann, Mooreberg, et al. 2019). This highlights the need for soil temperature and moisture sensors in deeper layers to be installed at field sites, particularly when measuring fluxes.

Though interannual variability is overpredicted, mean annual CH_4_ efflux is within 6% of the observed value. Observed and simulated CH_4_ efflux response to soil temperature showed a strong relationship, with the exception of the years the model underpredicted soil temperature. Methanogenesis did not take place at these lower temperatures and did not affect predictions of annual emissions, due to minimal winter fluxes (Euskirchen et al. 2024). The response of CH_4_ to soil moisture at 6 cm depth did not show a strong relationship with observed or simulated data. Deeper soil moisture measurements, where higher levels of methanogenesis are taking place, or longer term measurements of water table depth would be more appropriate to assess the response of this ecosystem to soil moisture changes. Nevertheless, structural uncertainty associated with additional thermal and hydrological process considerations is undoubtedly coupled to CH_4_ predictions.

We found positive correlations between methanogenesis‐related rate‐limiting constants and CH_4_ variables, and similarly, with RH rate‐limiting constants and RH variables. Both CH_4_ and RH had higher correlations with rate‐limiting constants associated with organic matter pools characterized by fast turn‐over, with more abundance in upper soil layers. CH_4_ was negatively correlated with the RH rate‐limiting constants, and RH was negatively correlated with the methanogenesis rate‐limiting constants, suggesting an inverse coupling between the two processes and strong dependence on C substrate. Burial constants, the rates of C transfer between shallower and deeper soil horizons, impacted CH_4_, RH, ALD, and WTD, although correlations varied. This highlighted the importance of C substrate availability as well as its distribution within the soil column. The fibric to humic burial constant had a strong positive correlation to ALD but a strong negative correlation to WTD. As more C moved from shallower fibric to deeper humic layers, ALD increased as insulation decreased, though WTD became shallower, which allowed methanogenesis to access the larger C pool. This aligns with correlations of thermal and hydraulic conductivities with WTD and CH_4_. This feedback affects CH_4_ transport pathway through movement of the WTD, modifying ebullition and changing the thickness of the oxic upper soil layers linked to oxidation and diffusion. The largest correlations for biophysical parameters were *n*factor_s_ and *ρ*_Snow,max_ both impacting output variables through modification of the thermal and hydrological regime particularly at the soil surface. Both *n*factors and *ρ*_Snow,max_, along with many other parameters are kept constant during typical simulations, though these properties may change in a warming climate, affecting predictions of future CH_4_ dynamics. Changes in vegetation composition and structure, as well as soil properties can impact *n*factors seasonally and interannually, which creates transient parameter uncertainty particularly if disturbances are integrated within models. Changes in *ρ*_Snow,max_ could be impacted by wind speed and direction as well as rain‐on‐snow events, which are increasingly common in the high latitudes. This finding suggests potential benefits in a dynamic parameterization approach, provided appropriate data is available to inform and validate model changes.

We modified summer and winter air temperature and precipitation input forcings and ran simulations for 1000 years to reach equilibrium under the modified climate using our calibrated (aerenchyma‐dominant) parameterization, representing our model response to long‐term stabilization following ecological change. We quantified the change from the baseline climate forcing using a sensitivity index. Temperature changes had larger effects than precipitation, suggesting changes of > ±15 mm may be required to elicit model feedback. Increases in air temperature had larger responses than decreases. As this site is on discontinuous permafrost, with a MAAT of −2.5°C (during pre‐industrial conditions), small increases in temperature may lead to destabilization of the thermal and hydrological regime resulting in a greater response. This mechanism is even more important for current conditions, with a MAAT of −0.8°C, where active degradation is evident at the site. There were strong relations between unfrozen soil C, ALD, WTD, CH_4_, and RH dynamics. CH_4_ fluxes were more sensitive to long‐term climate changes than RH. Warming air temperatures, particularly in the winter, increased ALD and unfrozen soil C. Similarly, increases in winter precipitation showed the impact of snow dynamics on permafrost stability (Jafarov et al. 2014). This was supported by high correlations with *ρ*_Snow,max_. CH_4_ efflux increased in response to warming but to a lesser extent at 3°C of warming due to ALD increases and soil C decreases. This was evident from the lowering of CH_4,Pool_. This again suggested sensitivity to soil C availability and distribution. Cooler air temperatures promoted C accumulation in deeper horizons, which promoted CH_4_ efflux. Nevertheless, the relationships between response and changes in input forcing suggest a dependence on the parameterization calibrated for the baseline climate. Dynamic parameter shifts may be required to capture ecosystem change at longer timescales, though this would require additional observational data (De Keersmaecker et al. 2014; Botkin and Sobel 1975; Pennekamp et al. 2018). There is additional structural uncertainty from the use of monthly input forcing data rather than daily or sub‐daily, which limits the accuracy in capturing rapid freeze–thaw or heavy precipitation events, which will impact CH_4_ pulse events. Though a pseudo daily time step is used by interpolation of monthly input forcings to artificially enhance the resolution in numerical solvers, maxima and minima are often missed. Though integration of higher resolution input forcings is desirable, they limit the applicability of the model for large‐area simulations. Hot moments remain challenging to capture due to observational data scarcity and the translation from fine‐ to coarse‐scale spatial processes.

Greatest projected CH_4_ emissions were calculated for ACCESS SSP3‐7.0 and MRI SSP2‐4.5. Though MRI SSP5‐8.5 had the most immediate response, increases in ALD and subsequently WTD reduced overall CH_4_ efflux due to soil drying (Voigt et al. 2023). If soil saturation was maintained, we would expect increased CH_4_ efflux throughout the century. We attributed higher CH_4_ efflux for lower SSPs to smaller temperature increases but higher precipitation in MRI. Less severe SSPs appeared to prolong methanogenesis and efflux through exposure of soil C while maintaining shallower WTDs, resulting in greater cumulative CH_4_ efflux. Predictions of NEE showed a C sink across all SSPs and ESMs. However, when including CH_4_, we predicted a C source for various years between 2035 and 2058. This resulted in a positive radiative forcing across all SSPs and ESMs, until the end of the century. Our predictions of NEE likely underestimate soil C emissions and overestimate the CO_2_ fertilization effect compared with observations due to known model biases (Euskirchen et al. 2024), suggesting this warming effect could be ~10% more pronounced. This also demonstrated a limitation in the calibration process whereby GPP and RECO (which are partitioned from NEE and not directly measured) are used but can be challenging to measure due to extreme day and night lengths in the high latitudes.

Projections with different emission pathway‐dominance demonstrated a large source of uncertainty. Diffusion‐dominant emission increased interannual variability, whereas ebullition‐dominant emission decreased interannual variability in predicted CH_4_ efflux. Though the diffusion‐dominant parameterization led to similar positive radiative forcing predictions as our final calibration (aerenchyma‐dominant) the ebullition‐dominant projections resulted in a negative mean radiative forcing or net cooling effect. This is particularly concerning due to the lack of measurements or techniques to fully partition CH_4_ emission pathways and could lead to diametrically opposed predictions. Further, the missing model hydrological dynamics (e.g., lateral water flux, surface water layers) could prevent soil drying increasing ebullitive fluxes. Though our combination of aerenchyma, diffusion, and ebullition are supported by analyzing the seasonal CH_4_ dynamics during the calibration process, changes to these processes over time given permafrost degradation and even ecosystem transitions are challenging to predict. Though our findings suggest a high‐level of uncertainty based on transport pathway dominance, further testing will be required to analyze the magnitude and direction of this effect across different locations and wetland types, exhibiting varied soil and vegetation properties, as well as hydrological dynamics.

The effect of net cooling given an ebullition‐dominant parameterization occurs because of relationships between temperature, moisture, and carbon substrate availability. Ebullition can only occur in soil layers below the water table which does not remain at the soil surface during the thawed season (Figure 3a). This leads to low emissions occurring directly from ebullition across parameterizations (Figure 4b) with mean annual emission lowest in the ebullition‐dominant simulation (Figure 6). This is despite the inclusion of an exponential function (Supporting Information: equation 6) allowing ebullition‐transported CH_4_ to be emitted to help improve predictions given spatial heterogeneity in microtopography and water table depth present in wetland ecosystems. Therefore, ebullition primarily redistributes CH_4_ from lower to upper soil layers. Unlike aerenchyma, which are able to bypass the upper aerobic layers, ebullition‐transported CH_4_ is largely released into these layers, increasing the CH_4_ concentration and CH_4_ oxidation prior to emission via diffusion. This effect reduces overall CH_4_ emissions when transport is dominated by ebullition. As air temperature increases in projected climate scenarios, the active layer and water table deepen, increasing the oxic zone and lowering future CH_4_ emissions compared to aerenchyma‐ or diffusion‐dominant parameterizations. This is likely to be different given fully inundated ecosystems such as marshes or wet tundra in coastal lowlands.

Recent model intercomparisons show variations in predicted CH_4_ emissions have been linked to differences in predicted seasonal and interannual variability, but also wetland extent (Melton et al. 2013; Bohn et al. 2015). Additional sources of uncertainty are specific to cold regions, with underpredictions of CH_4_ between September and May, and parameterization affecting freeze–thaw processes causing variability (Ito et al. 2023). Cold season dynamics and permafrost degradation are important for assessing the feedback from high latitudes to the global Earth system (Natali et al. 2019; Zhang et al. 2025). Forbrich et al. (2024) noted the need for improved biogeochemical and hydrological dynamics between different wetland classes (e.g., bogs, fens) combined with expansion of observational networks for variables such as WTD and CH_4_ concentrations will be important in reducing the discrepancies between bottom‐up and top‐down model predictions. Xu et al. (2016) found among 39 ESMs, prediction mismatch was due to differing representation of landcover characteristics and inundation dynamics, with improvement needed in validation of CH_4_ processes over depth and horizontal space. Further, differences in vegetation types and their rooting depths can lead to substantial variations in CH_4_ transport (Määttä and Malhotra 2024) as well as the role of microbial communities on methanogenesis and methanotrophy is essential, with their temperature sensitivity and C substrate availability greatly influencing fluxes (McClain et al. 2003). Salmon et al. (2022) also found predicted methanogenesis was sensitive to temperature and C substrate availability. Tilak et al. (2024) reviewed 16 wetland models, finding < 50% representing methanogenesis and oxidation, with less capturing transport processes. 25% Of the 16 models represented ebullition adequately. Ma et al. (2022) discussed parameter‐based equifinality, where different parameter sets produce the same results, which can lead to high uncertainty in model projections, describing improved ebullition predictions by using a bubble growth volume threshold approach constrained using CH_4_ concentration profiles.

## 5 Conclusion

Our results demonstrate the importance of thorough evaluation of sensitivities and uncertainties associated with model predictions of CH_4_ in prevalent yet heterogeneous boreal wetland ecosystems, and the limitations in long‐term projections under climate change scenarios. In particular, we highlight the sensitivity of our model processes to both the carbon substrate availability and distribution within the soil, both highly variable spatially and temporally. We find limitations to our predictions due to the representation of complex hydrological processes, which may further increase CH_4_ emissions by preventing soil drying as permafrost thaws. This study also has implications for other models predicting CH_4_, wetland and permafrost dynamics, with seasonality found to be important for accurate model calibration and impacts associated with the dominant transport pathway affecting the magnitude and direction of predicted radiative forcing under climate warming scenarios. Our findings suggest the importance of long‐term CH_4_ flux measurements with concentration profiles in the soil and developing techniques to measure the partitioning between different CH_4_ emission pathways, to better constrain our predictions of carbon dynamics in boreal wetlands under pronounced environmental change.

## Author Contributions

**Andrew Mullen:** writing – review and editing, writing – original draft, conceptualization. **Jennifer D. Watts:** conceptualization, writing – original draft, writing – review and editing. **Chu‐Chun Chang:** conceptualization, writing – original draft, writing – review and editing. **Joshua M. Rady:** conceptualization, writing – original draft, writing – review and editing. **Brendan M. Rogers:** conceptualization, methodology, supervision, project administration, writing – original draft, writing – review and editing, funding acquisition. **Benjamin C. Maglio:** conceptualization, methodology, investigation, validation, formal analysis, visualization, writing – original draft, writing – review and editing, software. **Valeria Briones:** writing – review and editing, resources, writing – original draft, conceptualization. **Ruth Rutter:** software, validation, writing – review and editing, writing – original draft, conceptualization. **Eugénie S. Euskirchen:** conceptualization, data curation, writing – original draft, writing – review and editing. **Colin Edgar:** data curation, visualization, writing – original draft, writing – review and editing. **Hélène Genet:** conceptualization, methodology, software, investigation, supervision, funding acquisition, project administration, writing – original draft, writing – review and editing. **Tobey Carman:** software, resources, writing – original draft, writing – review and editing. **Elchin E. Jafarov:** conceptualization, software, methodology, writing – original draft, writing – review and editing, project administration, supervision. **Susan M. Natali:** conceptualization, funding acquisition, writing – original draft, writing – review and editing, project administration.

## Funding

This work was supported by the TED Audacious Project (Permafrost Pathways), Google.org's Impact Challenge for Climate Innovation Program (Permafrost Discovery Gateway), and the Bonanza Creek Long Term Ecological Research Program, National Science Foundation grants (DEB‐0425328, DEB‐0724514, and DEB‐0830997), and the United States Geological Survey Climate R&D Program.

## Conflicts of Interest

The authors declare no conflicts of interest.

## Supporting information


**Figure S1:** Matrix showing correlation between biogeochemical parameters and output variables.

**Figure S2:** Matrix showing correlation between biophysical parameters and output variables.

**Figure S3:** Modifications to climate input forcings used in sensitivity analysis showing (a) summer and (b) winter air temperature, and (c) summer and (d) winter precipitation average monthly values as used in equilibrium run stage. The dashed lines represent baseline climate forcing data, with red and blue lines showing increases and decreases respectively.

**Figure S4:** Normalized sensitivity index for model output variables run for 1000 years under averaged climate with modifications made to air temperature and precipitation for summer and winter. Temperature and precipitation have been normalized to maximum response respectively. Variables are sorted by winter air temperature sensitivity index magnitude as this elicited the greatest sensitivity. Modifications to air temperature are shown on the top and precipitation on the bottom. Summer and winter changes are red and blue respectively. Acronyms are listed in the main text and within the Abbreviations table at the beginning of the Supporting Information.

**Figure S5:** Normalized sensitivity index response to winter and summer, air temperature and precipitation climate input forcings under stable state conditions. Sensitivity index is calculated using Equation (9) (Friend et al. 1993). Points represent the mean sensitivity index and the bars show the range between responses. Values are normalized to the maximum sensitivity index response for each variation in climate forcing shown and values are sorted by the absolute value of the mean calculated for changes in winter air temperature as this showed the largest response. The maximum sensitivity index is shown for each input forcing change.

**Figure S6:** Downscaled annual air temperature and precipitation forcing data from ACCESS and MRI for SSP1‐2.6, SSP2‐4.5, SSP3‐7.0, SSP5‐8.5 used for future projections.

**Figure S7:** Projected annual CH_4_ efflux and cumulative annual CH_4_ efflux under ACCESS and MRI ESMs and scenarios SSP1‐2.6, SSP2‐4.5, SSP3‐7.0, SSP5‐8.5 using an ebullition‐dominant parameterization.

**Figure S8:** Projected annual CH_4_ efflux cumulative annual CH_4_ efflux under ACCESS and MRI ESMs and scenarios SSP1‐2.6, SSP2‐4.5, SSP3‐7.0, SSP5‐8.5 using a diffusion‐dominant parameterization.

**Figure S9:** Projected active layer depth under scenarios SSP1‐2.6, SSP2‐4.5, SSP3‐7.0, SSP5‐8.5 for ACCESS and MRI ESMs. Mean and range of scenarios is shown by the solid line and shaded area respectively.

**Figure S10:** Projected water table depth under scenarios SSP1‐2.6, SSP2‐4.5, SSP3‐7.0, SSP5‐8.5 for ACCESS and MRI ESMs. Mean and range of scenarios is shown by the solid line and shaded area respectively.

**Figure S11:** Projected ecosystem respiration (RECO) under scenarios SSP1‐2.6, SSP2‐4.5, SSP3‐7.0, SSP5‐8.5 for ACCESS and MRI ESMs. Mean and range of scenarios is shown by the solid line and shaded area respectively.

**Figure S12:** Projected gross primary productivity (GPP) under scenarios SSP1‐2.6, SSP2‐4.5, SSP3‐7.0, SSP5‐8.5 for ACCESS and MRI ESMs. Mean and range of scenarios is shown by the solid line and shaded area respectively.

**Figure S13:** Projected net ecosystem exchange (NEE) under scenarios SSP1‐2.6, SSP2‐4.5, SSP3‐7.0, SSP5‐8.5 for ACCESS and MRI ESMs. Mean and range of scenarios is shown by the solid line and shaded area respectively.

**Table S1:** Parameters and ranges used in sensitivity analysis. Sample ranges were derived from literature sources where possible and cited next to the given range. *The *n*‐factor describes the ratio of ground‐surface temperature to air temperature (Kade et al. 2006).

## Data Availability

All associated data, scripts, and model code used or generated as part of this manuscript are available in Zenodo repository: DVM‐DOS‐TEM Thermokarst Bog CH_4_ Model (https://doi.org/10.5281/zenodo.18237274).
