# Supplementary figures and images for: Projected Methane Emissions From a Boreal Thermokarst Bog Are Sensitive to Carbon Substrate Availability, Distribution, and Transport Pathway Dominance
Source: Glob Chang Biol. 2026 Apr 28;32:e70880. doi: 10.1111/gcb.70880 (PMC13122443; doi:10.1111/gcb.70880)

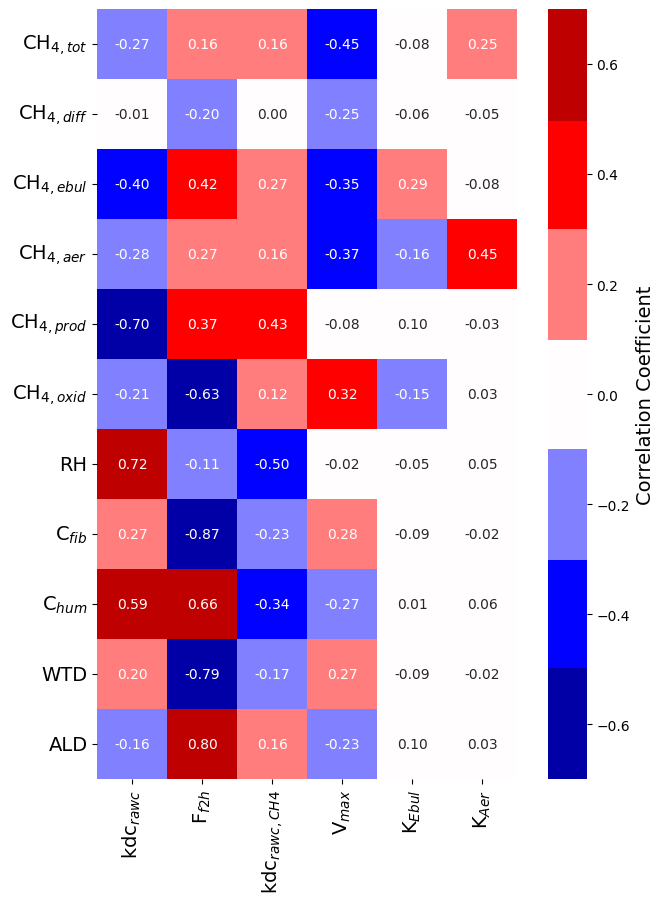

Supplement: Supplementary file 1 — Figure S1: Matrix showing correlation between biogeochemical parameters and output variables. [file GCB-32-e70880-s008.png]

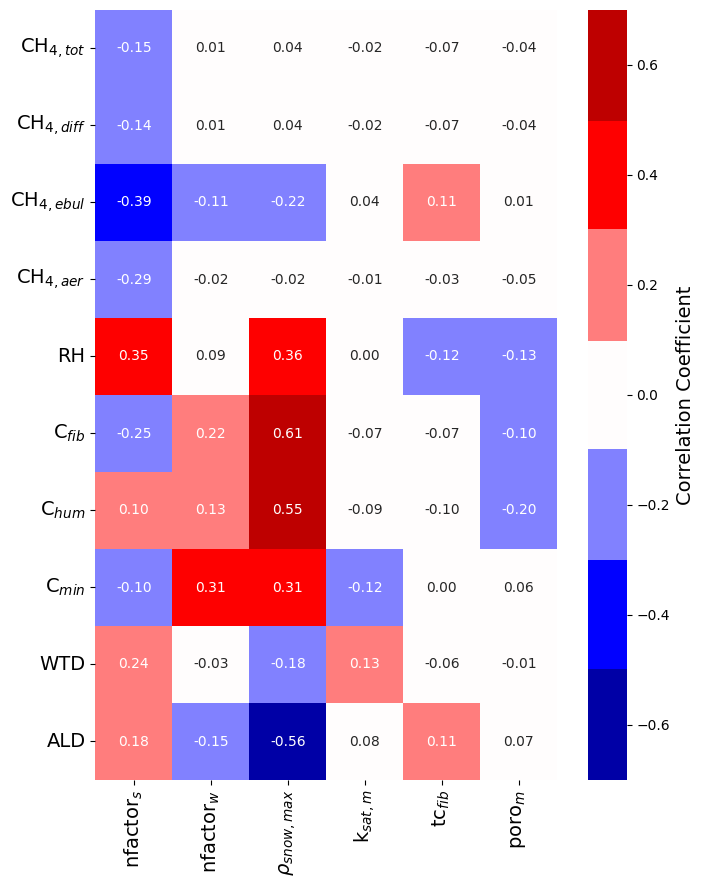

Supplement: Supplementary file 2 — Figure S2: Matrix showing correlation between biophysical parameters and output variables. [file GCB-32-e70880-s007.png]

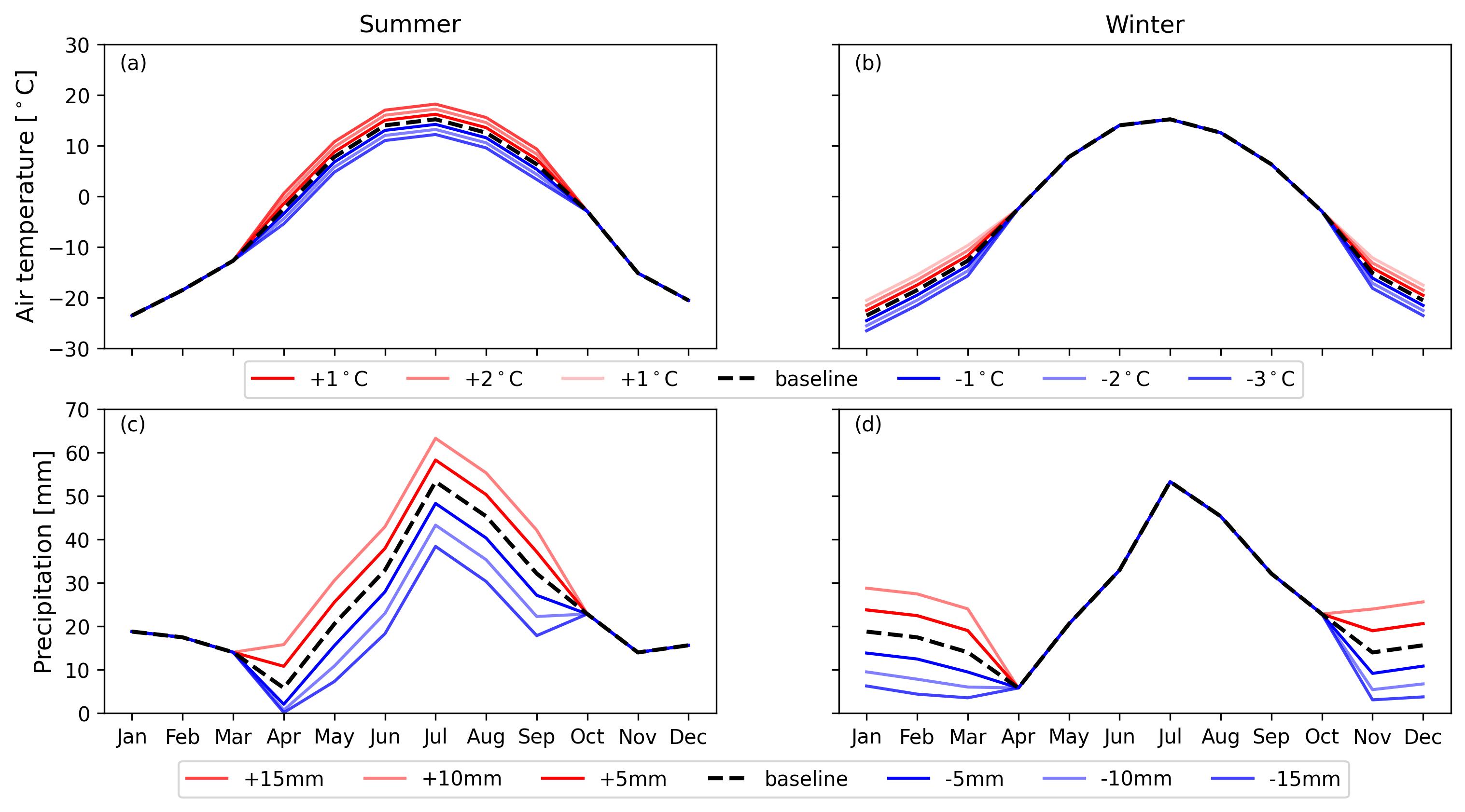

Supplement: Supplementary file 3 — Figure S3: Modifications to climate input forcings used in sensitivity analysis showing (a) summer and (b) winter air temperature, and (c) summer and (d) winter precipitation average monthly values as used in equilibrium run stage. The dashed lines represent baseline climate forcing data, with red and blue lines showing increases and decreases respectively. [file GCB-32-e70880-s012.jpg]

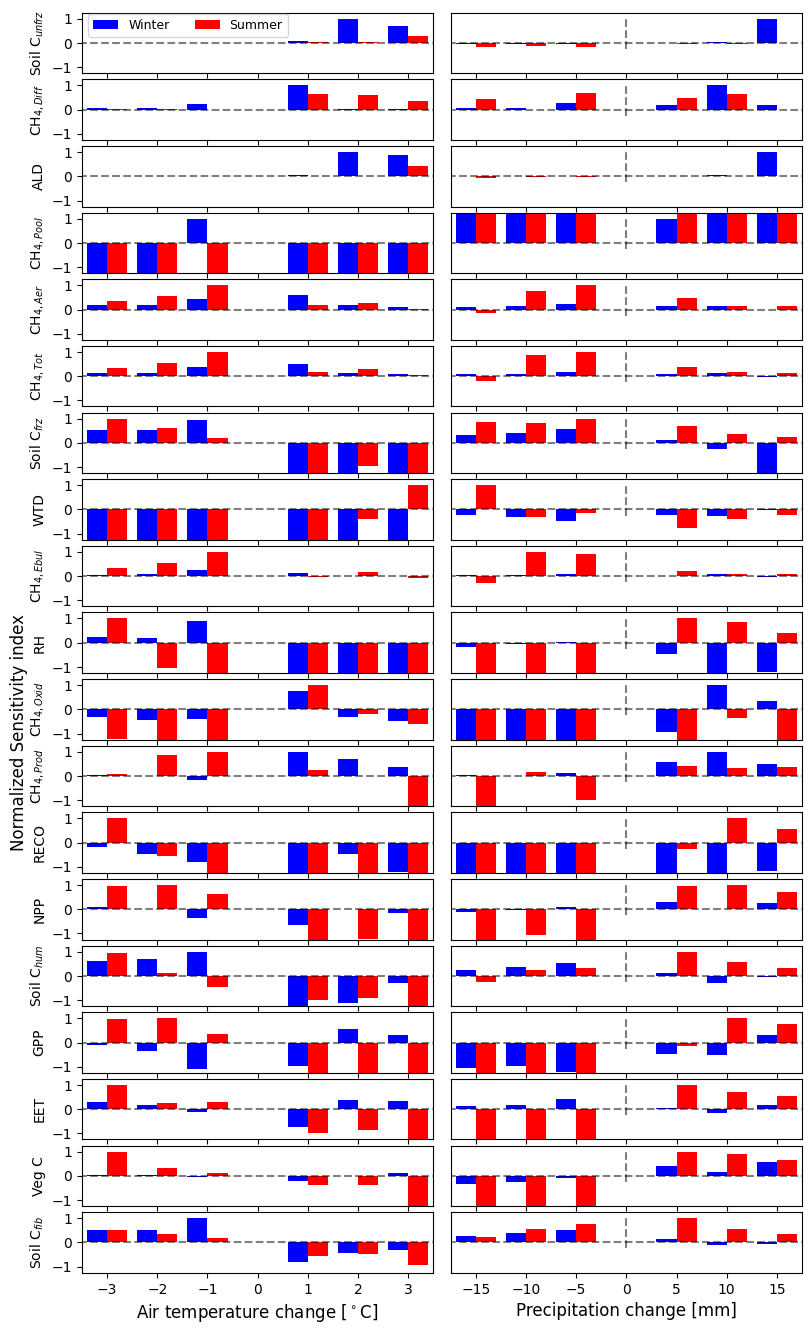

Supplement: Supplementary file 4 — Figure S4: Normalized sensitivity index for model output variables run for 1000 years under averaged climate with modifications made to air temperature and precipitation for summer and winter. Temperature and precipitation have been normalized to maximum response respectively. Variables are sorted by winter air temperature sensitivity index magnitude as this elicited the greatest sensitivity. Modifications to air temperature are shown on the top and precipitation on the bottom. Summer and winter changes are red and blue respectively. Acronyms are listed in the main text and within the Abbreviations table at the beginning of the Supporting Information. [file GCB-32-e70880-s009.png]

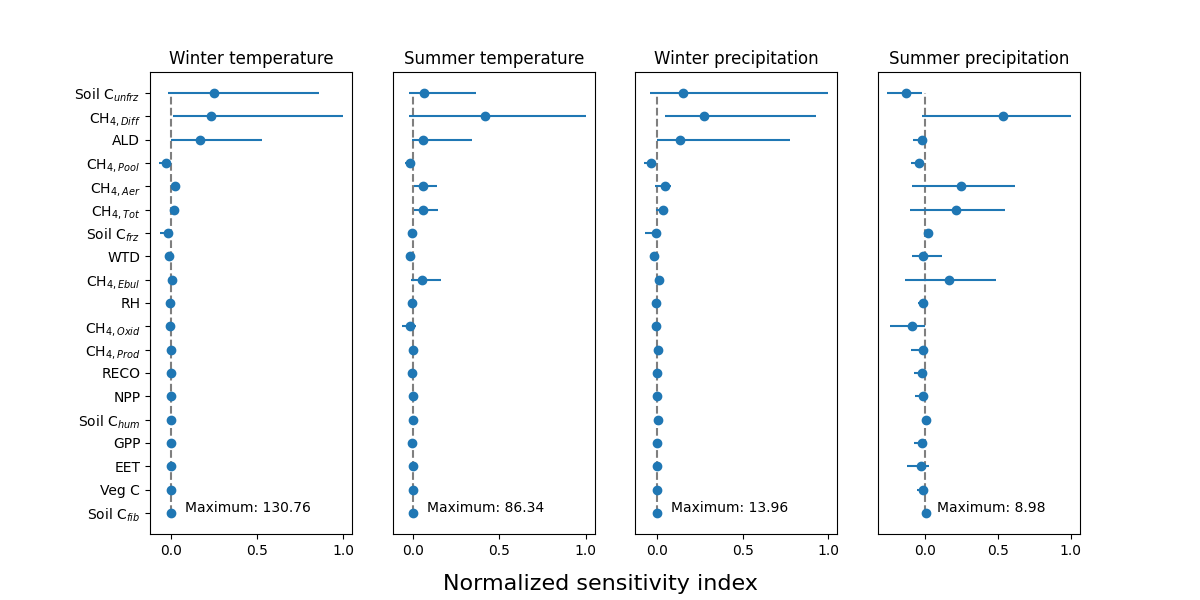

Supplement: Supplementary file 5 — Figure S5: Normalized sensitivity index response to winter and summer, air temperature and precipitation climate input forcings under stable state conditions. Sensitivity index is calculated using Equation (9) (Friend et al. 1993). Points represent the mean sensitivity index and the bars show the range between responses. Values are normalized to the maximum sensitivity index response for each variation in climate forcing shown and values are sorted by the absolute value of the mean calculated for changes in winter air temperature as this showed the largest response. The maximum sensitivity index is shown for each input forcing change. [file GCB-32-e70880-s004.png]

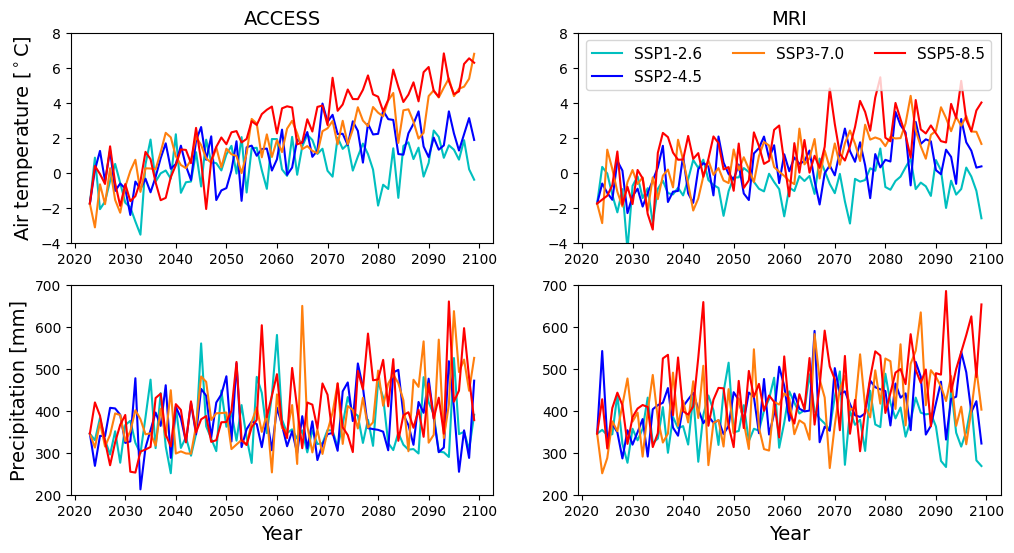

Supplement: Supplementary file 6 — Figure S6: Downscaled annual air temperature and precipitation forcing data from ACCESS and MRI for SSP1‐2.6, SSP2‐4.5, SSP3‐7.0, SSP5‐8.5 used for future projections. [file GCB-32-e70880-s006.png]

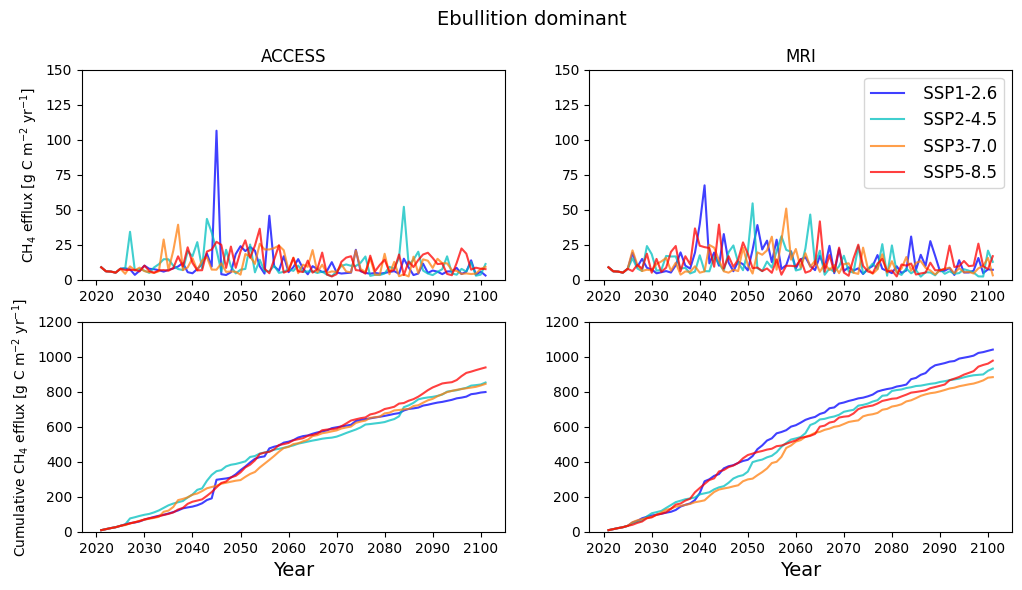

Supplement: Supplementary file 7 — Figure S7: Projected annual CH4 efflux and cumulative annual CH4 efflux under ACCESS and MRI ESMs and scenarios SSP1‐2.6, SSP2‐4.5, SSP3‐7.0, SSP5‐8.5 using an ebullition‐dominant parameterization. [file GCB-32-e70880-s013.png]

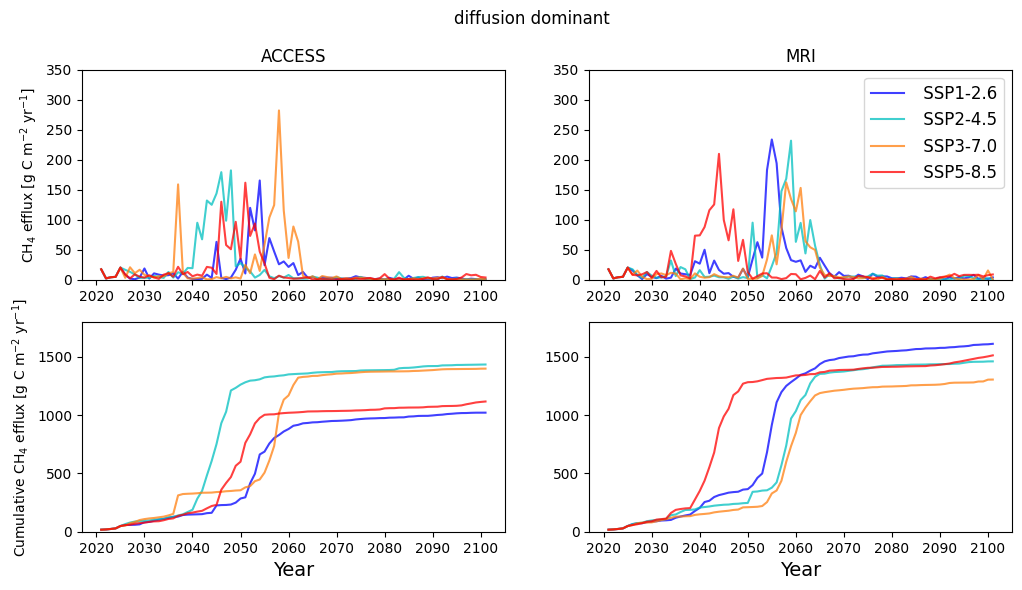

Supplement: Supplementary file 8 — Figure S8: Projected annual CH4 efflux cumulative annual CH4 efflux under ACCESS and MRI ESMs and scenarios SSP1‐2.6, SSP2‐4.5, SSP3‐7.0, SSP5‐8.5 using a diffusion‐dominant parameterization. [file GCB-32-e70880-s001.png]

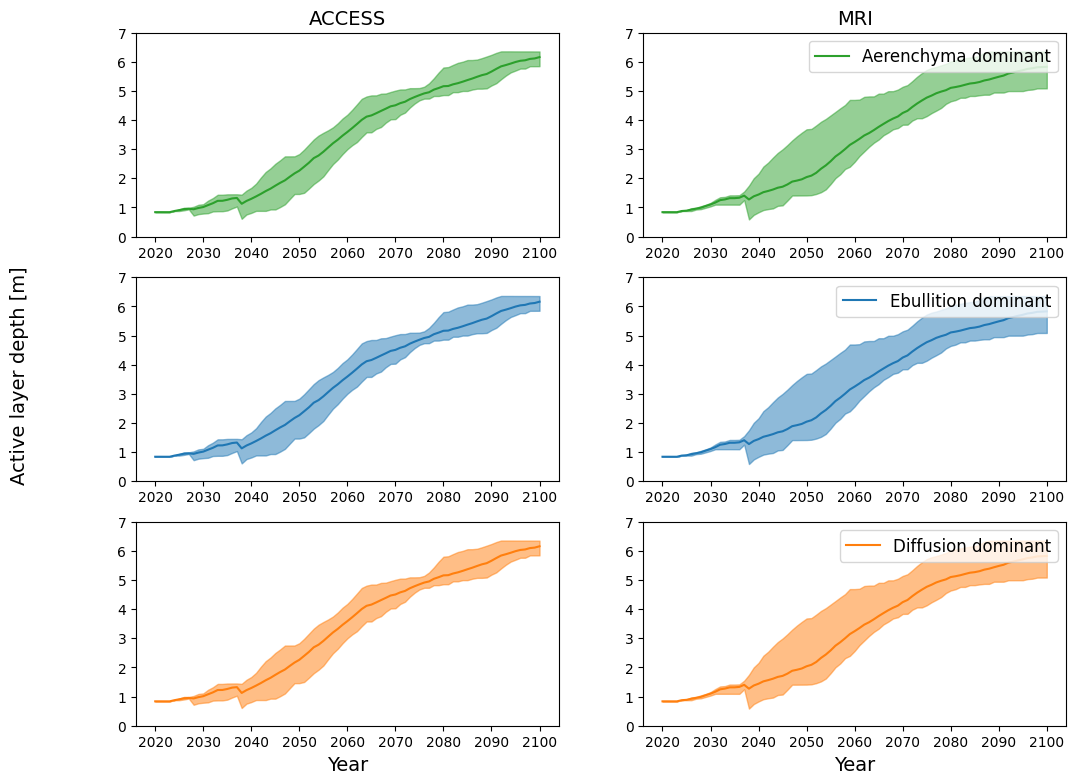

Supplement: Supplementary file 9 — Figure S9: Projected active layer depth under scenarios SSP1‐2.6, SSP2‐4.5, SSP3‐7.0, SSP5‐8.5 for ACCESS and MRI ESMs. Mean and range of scenarios is shown by the solid line and shaded area respectively. [file GCB-32-e70880-s002.png]

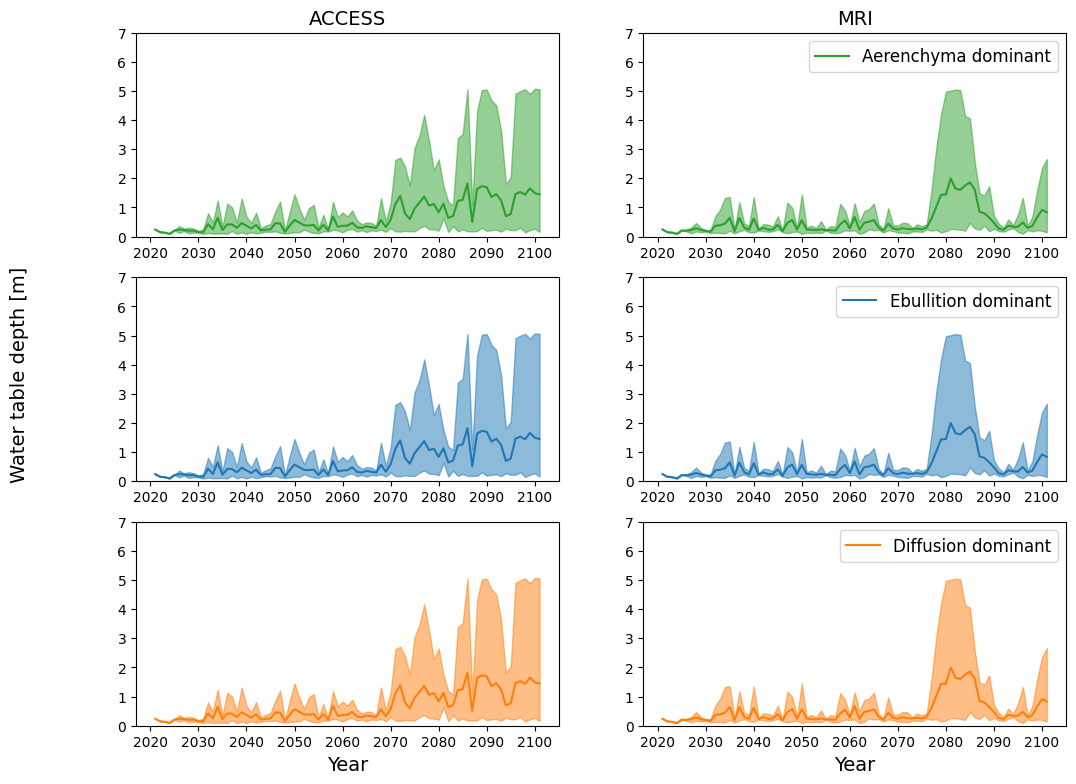

Supplement: Supplementary file 10 — Figure S10: Projected water table depth under scenarios SSP1‐2.6, SSP2‐4.5, SSP3‐7.0, SSP5‐8.5 for ACCESS and MRI ESMs. Mean and range of scenarios is shown by the solid line and shaded area respectively. [file GCB-32-e70880-s014.png]

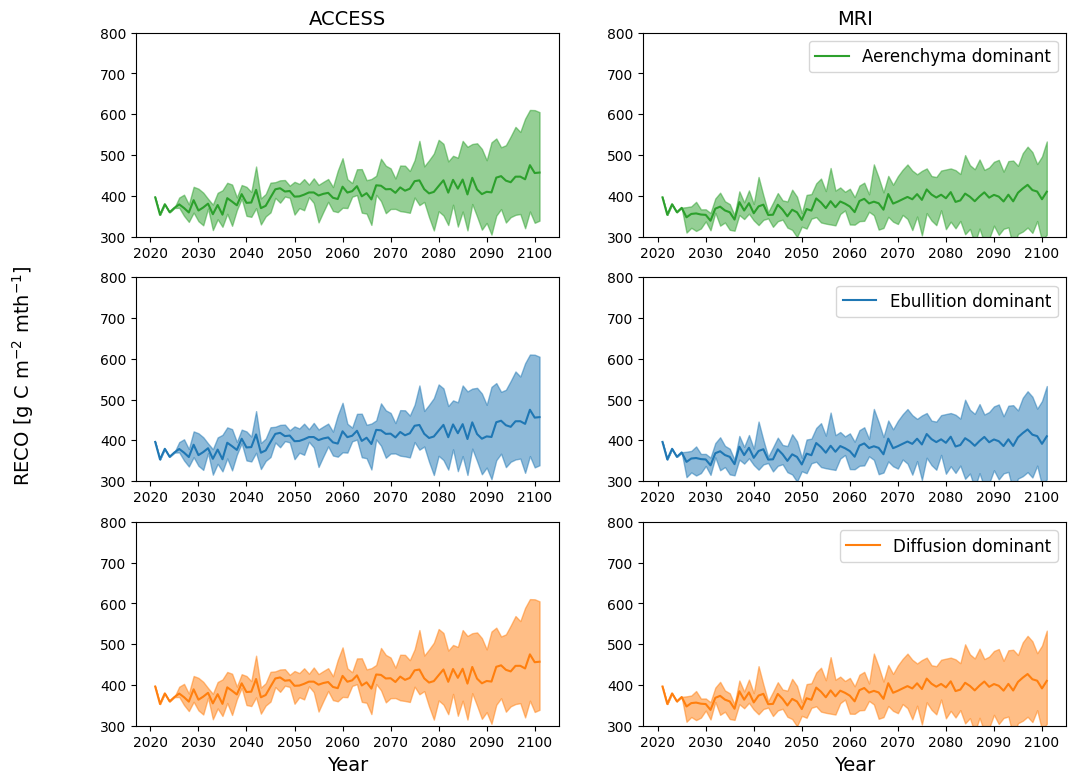

Supplement: Supplementary file 11 — Figure S11: Projected ecosystem respiration (RECO) under scenarios SSP1‐2.6, SSP2‐4.5, SSP3‐7.0, SSP5‐8.5 for ACCESS and MRI ESMs. Mean and range of scenarios is shown by the solid line and shaded area respectively. [file GCB-32-e70880-s003.png]

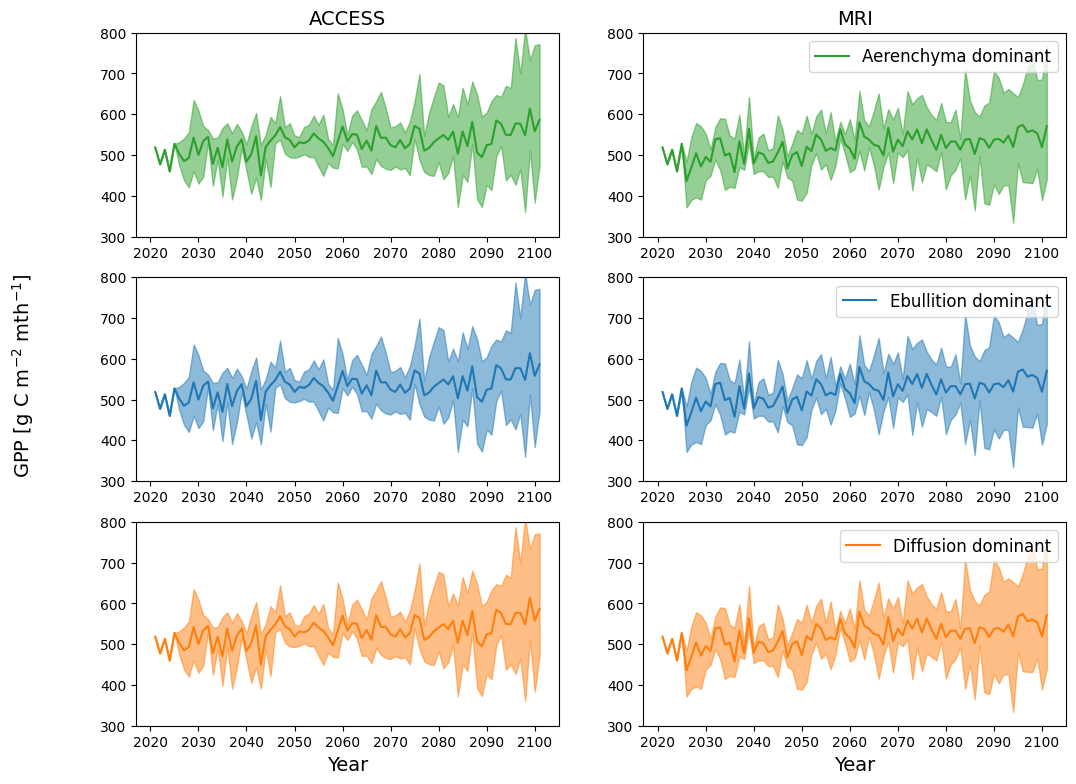

Supplement: Supplementary file 12 — Figure S12: Projected gross primary productivity (GPP) under scenarios SSP1‐2.6, SSP2‐4.5, SSP3‐7.0, SSP5‐8.5 for ACCESS and MRI ESMs. Mean and range of scenarios is shown by the solid line and shaded area respectively. [file GCB-32-e70880-s005.png]

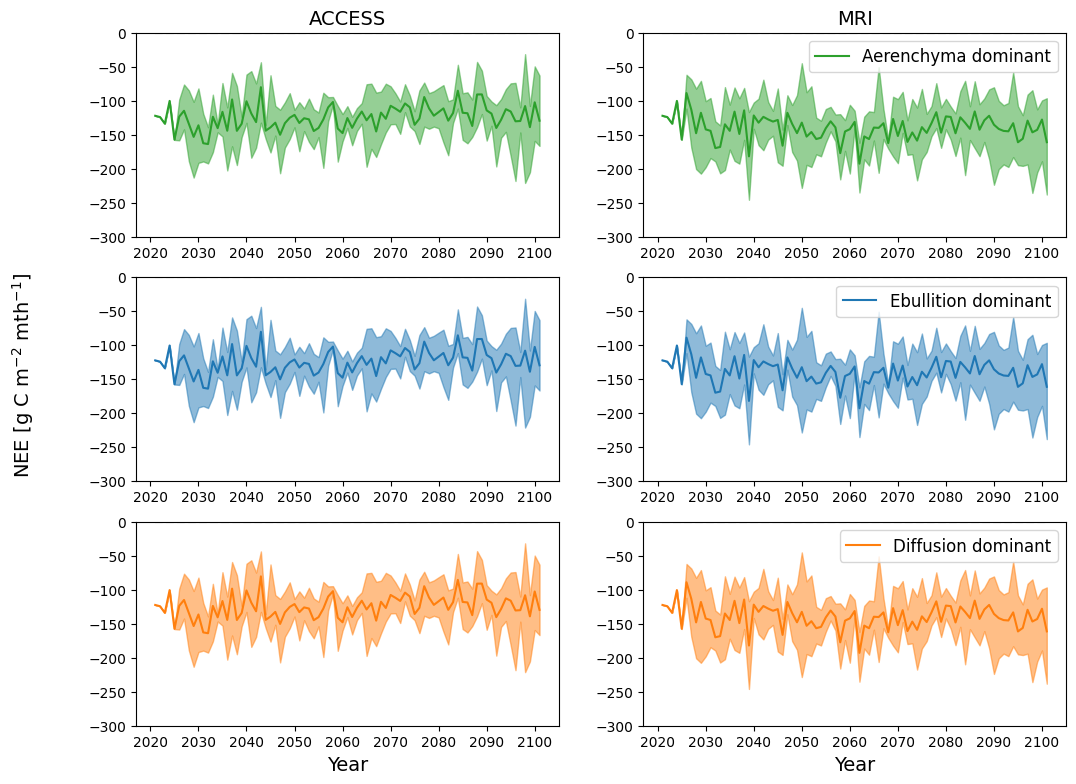

Supplement: Supplementary file 13 — Figure S13: Projected net ecosystem exchange (NEE) under scenarios SSP1‐2.6, SSP2‐4.5, SSP3‐7.0, SSP5‐8.5 for ACCESS and MRI ESMs. Mean and range of scenarios is shown by the solid line and shaded area respectively. [file GCB-32-e70880-s011.png]
