# Supplementary material for: Projected Methane Emissions From a Boreal Thermokarst Bog Are Sensitive to Carbon Substrate Availability, Distribution, and Transport Pathway Dominance
Source: Glob Chang Biol. 2026 Apr 28;32:e70880. doi: 10.1111/gcb.70880 (PMC13122443; doi:10.1111/gcb.70880)
Supplement: Supplementary file 14 — Table S1: Parameters and ranges used in sensitivity analysis. Sample ranges were derived from literature sources where possible and cited next to the given range. *The n‐factor describes the ratio of ground‐surface temperature to air temperature (Kade et al. 2006). [file GCB-32-e70880-s010.docx]

**Supplementary materials**

***Scientific Notation*** *- mathematical symbols used in order of introduction.*

| P_CH4_ | CH_4_ production (methanogenesis). |
| --- | --- |
| K_Prod, SOM_ | Methanogenesis rate-limiting constant by soil organic matter pool. |
| C_SOM, l_ | SOM C substrate by layer. |
| T_Prod, ref_ | Production-specific reference temperature. |
| T_degC, l_ | Temperature of the layer in ^○^C. |
| O_CH4_ | CH_4_ oxidation. |
| C_CH4, l_ | CH_4_ concentration by layer. |
| V_max_ | Michaelis-Menten kinetics parameter, maximum reaction rate at full saturation. |
| k_m_ | Michaelis-Menten kinetics parameter, CH_4_ concentration for half maximum reaction rate. |
| A_CH4_ | CH_4_ transport via aerenchyma. |
| K_Aer_ | Aerenchyma transport rate-limiting constant. |
| root_PFT, l_ | Fine root fraction by PFT and layer. |
| f(LAI_PFT_) | Function of PFT size relative to maximum leaf C [(Euskirchen et al. 2009)](https://www.zotero.org/google-docs/?V6PkZc). |
| Cap_PFT_ | CH_4_ transport capacity via aerenchyma by PFT. |
| E_CH4_ | CH_4_ transport via ebullition. |
| ***Scientific Notation*** *- mathematical symbols used in order of introduction.* | |
| K_ebul_ | Ebullitive transport rate-limiting constant. |
| S_M_ | Mass-based Bunsen solubility constant |
| S**_B_** | Bunsen solubility constant: ratio of dissolved CH_4_ to liquid water at atmospheric pressure. |
| T_degK, l_ | Temperature of the layer in K. |
| P_atm_ | Atmospheric pressure. |
| p | Total pressure. |
| z_l_ | Depth of the layer. |
| ф_l_ | Porosity of the layer. |
| f_Ebul_ | Fraction of ebullitive flux emitted from the soil column. |
| D_l_ | Diffusion coefficient of the layer. |
| γ | Reaction term: (P_CH4_-E_CH4_-O_CH4_-A_CH4_)dt |
| τ_l_ | Tortuosity of the layer. |
| D_0, l_ | Diffusion coefficient of CH_4_ in the layer (gas in water / air). |
| S | Sensitivity index ([(Friend et al. 1993)](https://www.zotero.org/google-docs/?zxnYuD). |
| v_a_ | Model output variable from altered input forcing. |
| v_bl_ | Model output variable from baseline input forcing. |
| ***Scientific Notation*** *- mathematical symbols used in order of introduction.* | |
| I_a_ | Altered input variable. |
| I_bl_ | Baseline input variable. |
| ***Abbreviations -*** *acronyms used in order of introduction* | |
| DVM-DOS-TEM | Dynamic Vegetation Model-Dynamics Organic Soil-Terrestrial Ecosystem Model. |
| PCCF | Permafrost carbon-climate feedback. |
| TBM | Terrestrial biosphere model. |
| ESM | Earth System model. |
| CMIP6 | Coupled Model Intercomparison Project phase 6. |
| SSP | Shared socioeconomic pathway. |
| RCP | Representative Concentration pathway. |
| ACCESS | Australian Community Climate and Earth System Simulator. |
| MRI | Meteorological Research Institute - ESM. |
| LTER | Long-term ecological research. |
| MAAT | Mean annual air temperature. |
| NEE | Net ecosystem exchange. |
| APEX | Alaska Peatland Experiment |
| ***Abbreviations -*** *acronyms used in order of introduction* | |
| CMT | Community type - a parameterization in DVM-DOS-TEM for a single gridcell. |
| PFT | Plant functional type. |
| TOPMODEL | A topography-based hydrological model. |
| NPP | Net primary productivity. |
| RECO | Ecosystem respiration. |
| GPP | Gross primary productivity. |
| RH / RHSOM | Heterotrophic respiration. |
| WTD | Water table depth. |
| ALD | Active layer depth. |
| VEGC | Vegetation carbon. |
| EET | Evapotranspiration. |

*CH_4_ flux model calculations*

Methanogenesis (P_CH4_) is calculated for all layers below the water table and interpolated within the layer containing the water table following Equation 1. P_CH4_ is the methanogenesis rate-limiting constant (K_Prod, SOM_), the C substrate in the layer (C_SOM, l_), and a function of temperature in ^○^C (degC; T_degC,l_) of that layer (l) following a Q10 relationship with a production-specific reference temperature (T_Prod, ref_) dependent on biome [(Zhuang et al. 2004)](https://www.zotero.org/google-docs/?aC4RNR).

$P_{CH4} = K_{Prod, SOM} C_{SOM, l} Q10^{(T_{degC,l}-T_{Prod, ref}/10)}$ (1)

C_SOM, l_ is separated by soil organic matter (SOM) pools: raw, active, physically-resistant, chemically-resistant for each layer. K_Prod, SOM_ is used for the calibration of P_CH4_ from that specific pool. Oxidation (O_CH4_) is calculated for all layers above the water table and interpolated within the layer containing the water table, using a Michaelis-Menten function shown in Equation 2. O_CH4_ depends on the CH_4_ concentration in a layer (C_CH4, l_). Similarly to P_CH4_, a function of temperature following a Q10 relationship is used with Q10 and reference temperature values specific to O_CH4_. V_max_ and k_m_ are kinetics parameters, describing the maximum reaction rate at full CH_4_ saturation and the CH_4_ concentration for which the reaction rate is half of the maximum, respectively.

$O_{CH4} =f(T_{degC,l})\frac{k_{m}C_{CH4,l}}{V_{max}+C_{CH4,l}}$ (2)

Transport of CH_4_ through aerenchyma (A_CH4_) is calculated by PFT and by soil layer using Equation 3. K_Aer_ is a rate limiting constant. root_PFT, l_ is the fine root fraction for a given PFT in a given soil layer. f(LAI_PFT_) is a function of the PFT size controlled by a foliage fraction relative to the maximum leaf C [(Euskirchen et al. 2009)](https://www.zotero.org/google-docs/?imLQVp). Cap_PFT_ is a PFT-specific transport capacity fraction (e.g. 80-90% of available CH_4_ is transported in aerenchymatous plants, i.e. *Carex*). 50% of aerenchyma transport is oxidized for a given soil layer while the remaining CH_4_ escapes the soil column as efflux (opposed to uptake).

${A_{CH4}} =K_{Aer}root_{PFT, l} f(LAI_{PFT}){Cap}_{PFT}C_{CH4, l}$ (3)

Layers are looped from bottom to top, with ebullition calculated prior to the processes already mentioned. This allows CH_4_ above a temperature-dependent concentration threshold in a given layer to be redistributed to the layer containing the water table (provided the layer is not completely frozen) before calculation of individual layer processes requiring updated CH_4_ concentration. A single-substance (tracking only a single gas, e.g. CH_4_), temperature-dependent threshold function is used to calculate the CH_4_ concentration above which gas is released through ebullition in Equation 4.

$E_{CH4} = K_{Ebul}(C_{CH4,l}-S_{M})$ (4)

Ebullition (E_CH4_) is calculated as the difference between C_CH4, l_ and the mass-based Bunsen Solubility Constant (S_M_, the concentration threshold above which bubbles begin to form) multiplied by a calibrated rate limiting constant (K_Ebul_), which may be used to correctly partition flux pathways if there is sufficient data from a site to do so. S_M_ is calculated based on a relationship defined by observations of dissolved CH_4_ at given temperatures [(Yamamoto et al. 2002)](https://www.zotero.org/google-docs/?dK9poo) shown in Equation 5.

$S_{B}= 0.05708-0.001545T_{degC} +0.00002069{T_{degC}}^{2}$ (5)

S_B_ is the volume ratio of dissolved CH_4_ gas to liquid water at atmospheric pressure at temperature in ^၀^C. S_M_ is found by applying the Ideal Gas Law, n=pV/RT_degK_ (with pressure p, volume V, temperature in degrees Kelvin T_degK_, and the ideal gas constant R). V is substituted for S_B_ and p is the sum of atmospheric and hydrostatic pressure (P_atm_+ρgz) [(Wania et al. 2010)](https://www.zotero.org/google-docs/?AZ82MT). If the water table is at the soil surface, calculated ebullition is fully considered efflux. If the water table is not at the soil surface, a fraction of the CH_4_ is redistributed from its corresponding layer and added to the layer containing the water table representing ebullitive movement within the soil column. Also, when the water table is not at the soil surface, an exponential function (Equation 6) is used to calculate a fraction of this ebullitive to leave the soil column as efflux depending on the depth (z_l_) and the porosity (ф_l_) of the layer. This allows control over the episodic emissions across larger cell scales (>1 km^2^)_,_ representing variability in water table depth and heterogeneity in soil surface microtopography (with hummocks and depressions common in peatlands). Here, f_Ebul_ represents the fraction of ebullitive flux in a given layer which exits the soil column. Observations show that bubbles move at high speeds through porous soils such as peat (Scheffer and Schachtschabel, 1982; Shafer and Zare, 1991) and flux occurs in the upper 90% of peat layers [(Klapstein et al. 2014)](https://www.zotero.org/google-docs/?3DW5r3) though there is associated heterogeneity [(Korrensalo et al. 2018)](https://www.zotero.org/google-docs/?nD8Ir0).

$f_{Ebul}=e^{-(z_{l}/\phi_{l})}$ (6)

Molecular diffusion is calculated once other fluxes are known. Diffusion of CH_4_ including efflux and uptake at the soil surface is calculated by solving the reaction-diffusion Equation (7). The rate of change in CH_4_ concentration is determined by the second derivative of CH_4_ concentration with respect to position in the soil column multiplied by a gas-in-soil diffusion coefficient (D_l_) and summed with a reaction term (γ).

$\frac{\partial C_{CH4}}{\partial t}=D_{l}\frac{\partial^{2}C_{CH4}}{\partial x^{2}}+\gamma$ (7)

D is calculated in Equation 8 for each soil layer. γ is the sum of positive and negative fluxes P_CH4_ and E_CH4_ (ebullitive efflux only as redistribution is already calculated), O_CH4_, and A_CH4_ respectively multiplied by the model time step (dt; which converts fluxes to a pool of CH_4_ change). Hence, γ=(P_CH4_ - E_CH4_ - O_CH4_ - A_CH4_)dt. D is calculated (Equation 8) from the diffusion coefficient of CH_4_ (D_0_) in either water, air or an interpolation when the layer contains the water table. τ_l_ is the soil tortuosity in the layer, describing the movement of gas through pores. τ_l_ is calculated as 0.66 θ (θ/ϕ)^3^ [(Moldrup et al. 1997)](https://www.zotero.org/google-docs/?ApibKo) following the results of a comparison of six tortuosity models [(Pingintha et al. 2010)](https://www.zotero.org/google-docs/?qVkQiG). θ is either the volumetric water or air content.

$D_{l}=D_{0,l}\tau_{l}\left( \frac{T_{degK,l}}{293.15} \right)^{1.75}$ (8)

The reaction-diffusion equation was discretized using the Crank-Nicolson method [(Crank and Nicolson 1947)](https://www.zotero.org/google-docs/?D3pTU6) and solved using the tridiagonal matrix algorithm [(Thomas 2013)](https://www.zotero.org/google-docs/?qD2UYY). These techniques require population of a matrix, which is done for all internal nodes during the preceding flux calculations, and subsequently the external nodes (or boundary conditions) are imposed, prior to solving. For gas diffusion at the top of the soil column, we assume a Dirichlet (open) boundary condition that varies with an atmospheric CH_4_ concentration input forcing, updated yearly based on historical or projected data. At the base of the soil column, a Neumann (closed) boundary condition specifies a zero change in CH_4_ concentration, assuming there is no transmission through bedrock.

***Table. S1.*** *Parameters and ranges used in sensitivity analysis. Sample ranges were derived from literature sources where possible and cited next to the given range. *The n-factor describes the ratio of ground-surface temperature to air temperature* [*(Kade et al. 2006)*](https://www.zotero.org/google-docs/?AxFQCo)*.*

| ***Parameter*** | ***Description*** | ***Unit*** | ***Sampled Range (reference)*** |
| --- | --- | --- | --- |
| kdc_rawc_ | Heterotrophic respiration rate constant for raw C pool. | [g g^-1^hr^-1^] | 0.05-0.35 |
| kdc_soma_ | Heterotrophic respiration rate constant for active C pool. | [g g^-1^hr^-1^] | 0.01-0.05 |
| kdc_sompr_ | Heterotrophic respiration rate constant for physically resistant C pool. | [g g^-1^hr^-1^] | 2x10^-5^-8x10^-5^ |
| kdc_somcr_ | Heterotrophic respiration rate constant for chemically resistant C pool. | [g g^-1^hr^-1^] | 1x10^-8^-2x10^-8^ |
| F_f2h_ | Fibric to humic C burial coefficient. |  | 0.5-1.0 |
| F_h2m_ | Humic to mineral C burial coefficient. |  | 0.5-1.0 |
| kdc_rawc, CH4_ | Methanogenesis rate constant for raw C pool. | [g g^-1^hr^-1^] | 2x10^-5^-6x10^-5^ |
| ***Parameter*** | ***Description*** | ***Unit*** | ***Sampled Range (reference)*** |
| kdc_soma, CH4_ | Methanogenesis rate constant for active C pool. | [g g^-1^hr^-1^] | 3x10^-6^-9x10^-6^ |
| kdc_sompr, CH4_ | Methanogenesis rate constant for physically resistant C pool. | [g g^-1^hr^-1^] | 2.5x10^-8^-7.5x10^-8^ |
| kdc_somcr, CH4_ | Methanogenesis rate constant for chemically resistant C pool. | [g g^-1^hr^-1^] | 2x10^-10^-6x10^-10^ |
| K_Ebul_ | Ebullition rate constant. | [g g^-1^hr^-1^] | 0.0001-0.001 [(Zhuang et al. 2004; Fan et al. 2013)](https://www.zotero.org/google-docs/?VP9fU8) |
| K_Aer_ | Aerenchyma transport rate constant. | [g g^-1^hr^-1^] | 0.001-0.006 [(Zhuang et al. 2004; Fan et al. 2013)](https://www.zotero.org/google-docs/?NXpZ5g) |
| K_m_ | Michaelis-Menten (MM) kinetics parameter (CH_4_ concentration when oxidation rate is half the maximum). | [μmol L^-1^] | 3-5 [(Walter and Heimann 2000)](https://www.zotero.org/google-docs/?FKZsNk) |
| V_max_ | Michaelis-Menten kinetics parameter (maximum oxidation rate for full CH_4_ saturation). | [μmol L^-1^ hr^-1^] | 5-50 [(Walter and Heimann 2000)](https://www.zotero.org/google-docs/?W88LTl) |
| ***Parameter*** | ***Description*** | ***Unit*** | ***Sampled Range (reference)*** |
| nfactor_s_ | Summer n-factor*. | [] | 0.2-2 [(Kade et al. 2006; Klene et al. 2001)](https://www.zotero.org/google-docs/?7DL1AA) |
| nfactor_w_ | Winter n-factor. | [] | 0.4-1.0 [(Kade et al. 2006; Klene et al. 2001)](https://www.zotero.org/google-docs/?wWgA2I) |
| ρ_Snow, max_ | Maximum snow density. | [kgm^-3^] | 100-800 [(Domine et al. 2016; Muskett 2012; Gerland et al. 1999)](https://www.zotero.org/google-docs/?JHgXEb) |
| k_sat, m_ | Saturated hydraulic conductivity of moss. | [mm/s] | 0.0002-30 [(Ekici et al. 2015; Letts et al. 2000; Liu et al. 2019)](https://www.zotero.org/google-docs/?Sz613m) |
| k_sat, f_ | Saturated hydraulic conductivity of fibric organic soil. | [mm/s] | 0.0002-30 [(Ekici et al. 2015; Letts et al. 2000; Liu et al. 2019)](https://www.zotero.org/google-docs/?HsVn8q) |
| k_sat, h_ | Saturated hydraulic conductivity of humic organic soil. | [mm/s] | 0.00004-2.01 [(Ekici et al. 2015; Letts et al. 2000; Liu et al. 2019)](https://www.zotero.org/google-docs/?2ZKh3F) |
| tc_m_ | Thermal conductivity of moss. | [Wm^-1^K^-1^] | 0.005-0.5 [(Jiang et al. 2015; Ekici et al. 2015; O’Donnell et al. 2009)](https://www.zotero.org/google-docs/?h9y5us) |
| ***Parameter*** | ***Description*** | ***Unit*** | ***Sampled Range (reference)*** |
| tc_f_ | Thermal conductivity of fibric organic soil. | [Wm^-1^K^-1^] | 0.005-0.5 [(Jiang et al. 2015; Ekici et al. 2015; O’Donnell et al. 2009)](https://www.zotero.org/google-docs/?D37kh3) |
| tc_h_ | Thermal conductivity of humic organic soil. | [Wm^-1^K^-1^] | 0.02-2.0 [(Jiang et al. 2015; Ekici et al. 2015; O’Donnell et al. 2009)](https://www.zotero.org/google-docs/?jQWoff) |
| poro_m_ | Porosity of moss. | [m^3^m^-3^] | 0.85-0.99 [(O’Donnell et al. 2009)](https://www.zotero.org/google-docs/?6Wt3HK) |
| poro_f_ | Porosity of fibric organic soil. | [m^3^m^-3^] | 0.85-0.99 [(O’Donnell et al. 2009)](https://www.zotero.org/google-docs/?4A4aoV) |
| poro_f_ | Porosity of humic organic soil. | [m^3^m^-3^] | 0.7-0.9 [(O’Donnell et al. 2009)](https://www.zotero.org/google-docs/?E07LK4) |

**Fig. S1.** Matrix showing correlation between biogeochemical parameters and output variables.

**Fig. S2.** Matrix showing correlation between biophysical parameters and output variables.

**Fig. S3.** Modifications to climate input forcings used in sensitivity analysis showing (a) summer and (b) winter air temperature, and (c) summer and (d) winter precipitation average monthly values as used in equilibrium run stage. The dashed lines represent baseline climate forcing data, with red and blue lines showing increases and decreases respectively.

**Fig. S4.** Normalized sensitivity index for model output variables run for 1000 years under averaged climate with modifications made to air temperature and precipitation for summer and winter. Temperature and precipitation have been normalized to maximum response respectively. Variables are sorted by winter air temperature sensitivity index magnitude as this elicited the greatest sensitivity. Modifications to air temperature are shown on the top and precipitation on the bottom. Summer and winter changes are red and blue respectively. Acronyms are listed in the main text and within the Abbreviations table at the beginning of the supplementary material.

**Figure. S5.** Normalized sensitivity index response to winter and summer, air temperature and precipitation climate input forcings under stable state conditions. Sensitivity index is calculated using equation 9 [(Friend et al. 1993)](https://www.zotero.org/google-docs/?6IoPBu). Points represent the mean sensitivity index and the bars show the range between responses. Values are normalized to the maximum sensitivity index response for each variation in climate forcing shown and values are sorted by the absolute value of the mean calculated for changes in winter air temperature as this showed the largest response. The maximum sensitivity index is shown for each input forcing change.

**Fig. S6.** Downscaled annual air temperature and precipitation forcing data from ACCESS and MRI for SSP1-2.6, SSP2-4.5, SSP3-7.0, SSP5-8.5 used for future projections.

**Fig. S7.** Projected annual CH_4_ efflux and cumulative annual CH_4_ efflux under ACCESS and MRI ESMs and scenarios SSP1-2.6, SSP2-4.5, SSP3-7.0, SSP5-8.5 using an ebullition-dominant parameterization.

**Fig. S8.** Projected annual CH_4_ efflux cumulative annual CH_4_ efflux under ACCESS and MRI ESMs and scenarios SSP1-2.6, SSP2-4.5, SSP3-7.0, SSP5-8.5 using a diffusion-dominant parameterization.

Fig. S9-13 show the projected responses of ALD, WTD, RECO, GPP, and NEE delineated by transport pathway-dominant (aerenchyma, ebullition, diffusion) parameterizations and by ESM (ACCESS and MRI). Projections show the mean and range across projected scenarios (SSP1-2.6, SSP2-4.5, SSP3-7.0, SSP5-8.5) by solid lines and shaded areas respectively. Projected responses do not vary significantly among CH_4_ transport pathway dominance. This is because we maintain C stocks (at 89,635 gCm^-2^ at the beginning of the historical simulation period, 1901-01-01) and CO_2_-related fluxes (i.e. GPP, RECO; as shown in Fig. 8a) to provide consistency in this model experiment. Hence, temporal variations are driven primarily by projected climate and not by variations in CH_4_ transport pathway parameterizations, though thermal, hydrological, and carbon substrate changes over time feedback upon CH_4_ flux dynamics, as shown by parameter (Fig. S1 and S2) and input (Fig. S4 and S5) sensitivity analyses.

**Fig. S9.** Projected active layer depth under scenarios SSP1-2.6, SSP2-4.5, SSP3-7.0, SSP5-8.5 for ACCESS and MRI ESMs. Mean and range of scenarios is shown by the solid line and shaded area respectively.

**Fig. S10.** Projected water table depth under scenarios SSP1-2.6, SSP2-4.5, SSP3-7.0, SSP5-8.5 for ACCESS and MRI ESMs. Mean and range of scenarios is shown by the solid line and shaded area respectively.

**Fig. S11.** Projected ecosystem respiration (RECO) under scenarios SSP1-2.6, SSP2-4.5, SSP3-7.0, SSP5-8.5 for ACCESS and MRI ESMs. Mean and range of scenarios is shown by the solid line and shaded area respectively.

**Fig. S12.** Projected gross primary productivity (GPP) under scenarios SSP1-2.6, SSP2-4.5, SSP3-7.0, SSP5-8.5 for ACCESS and MRI ESMs. Mean and range of scenarios is shown by the solid line and shaded area respectively.

**Fig. S13.** Projected net ecosystem exchange (NEE) under scenarios SSP1-2.6, SSP2-4.5, SSP3-7.0, SSP5-8.5 for ACCESS and MRI ESMs. Mean and range of scenarios is shown by the solid line and shaded area respectively.
